# *In Vitro* Assembly of Multiple DNA Fragments Using Successive Hybridization

**DOI:** 10.1371/journal.pone.0030267

**Published:** 2012-01-26

**Authors:** Xinglin Jiang, Jianming Yang, Haibo Zhang, Huibin Zou, Cong Wang, Mo Xian

**Affiliations:** Biomaterials Center, Qingdao Institute of Bioenergy and Bioprocess Technology, Chinese Academy of Sciences, Qingdao, China; Inserm U869, France

## Abstract

Construction of recombinant DNA from multiple fragments is widely required in molecular biology, especially for synthetic biology purposes. Here we describe a new method, successive hybridization assembling (SHA) which can rapidly do this in a single reaction *in vitro*. In SHA, DNA fragments are prepared to overlap one after another, so after simple denaturation-renaturation treatment they hybridize in a successive manner and thereby assemble into a recombinant molecule. In contrast to traditional methods, SHA eliminates the need for restriction enzymes, DNA ligases and recombinases, and is sequence-independent. We first demonstrated its feasibility by constructing plasmids from 4, 6 and 8 fragments with high efficiencies, and then applied it to constructing a customized vector and two artificial pathways. As SHA is robust, easy to use and can tolerate repeat sequences, we expect it to be a powerful tool in synthetic biology.

## Introduction

Modifying organisms at the molecular level is the main base for the modern biotechnology, in which people often need to incorporate two or more DNA fragments into a larger construct. Alongside the traditional restriction enzyme-based assembly, a variety of methods have been developed for this task in the last 40 years, which were systemically reviewed in a recent article by Ellis et al. [1]. The most recent and interesting development would be the new assembly strategy exemplified by SLIC which can assemble multiple fragments simultaneously and sequence-independently by harnessing *in vitro* homologous recombination. A single SLIC reaction could assemble a 7.5 kb plasmid from ten PCR fragments with 40 bp homologous ends (also called overlaps) [2]. A similar method used in constructing the first artificial organism, assembled the 583 kb *Mycoplasma genitalium* genome from four fragments with 80–257 bp homologous ends [3]. However these methods require high-quality DNA substrates and enzymes, thus DNA purification and reaction buffer switch are usually needed. Furthermore, faulty recombinations resulted from mismatching at overhangs were observed and got worse as the number of fragments increased [2], [3].

To address these limitations, we developed here a novel mechanism-based method, successive hybridization assembling (SHA), which does not include complex cut-ligate steps or specific recombination enzymes, and as found in our study, has outstanding performances regarding to simplicity, efficiency, and resistance to sequence repeats.

## Results

### Method design

**Figure 1:**
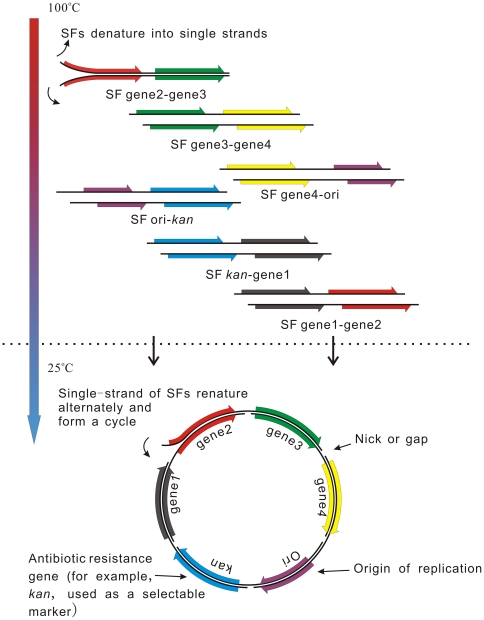
Scheme of SHA (take a 6-SF assembling for example). To construct the final plasmid from the six starting materials (gene 1–4, replication origin and a selectable marker), SFs are first prepared by linking every two materials together, usually suing overlap-extension PCR (OE-PCR). So as shown in this figure, every SF has its 3′-half overlapped with the 5′-half of the next SF and the 5′-half of the first SF overlaps with the 3′-half of the last SF. A mixture of these SFs was denatured at 100°C to free all single strands. When it cools back down to room temperature, annealing between the overlaps would assemble the single strands one after another into a cycle which can be further repaired into double-stranded, closed circular molecule after transformation into the cells.

In traditional DNA-DNA hybridization, for example in southern blotting, two different DNA pieces are recombined together with their complementary regions. We reasoned that with more hybridization events occurring, more DNA pieces can be recombined together for construction purpose. Based on this assumption we designed our SHA method. As shown in **Fig. 1** every substrate fragment (SF) is designed to have its 3′-half overlapped with the 5′-half of the next SF. As these overlaps (halves) can cover about 1/3 to 2/3 of each SF, after denaturation and renaturation, there are good chances for inter-fragment hybridizations. Then a circular plasmid can form and be ready for transformation. Nicks and gaps in it can be repaired in the bacteria cells.

**Figure 2:**
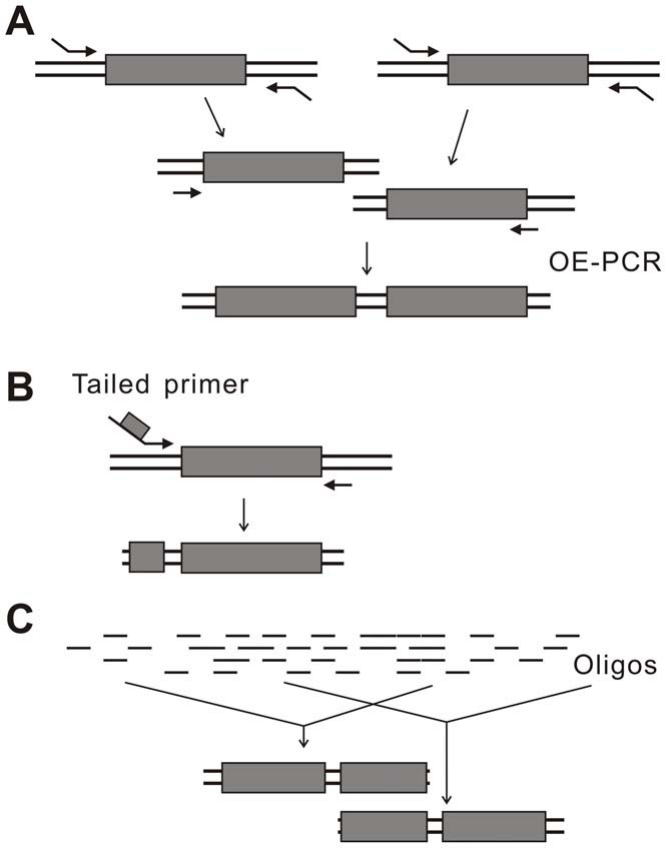
General methods for SF preparation. (**A**) Two elements are joined by OE-PCR. (B) One short element is added to the 5′-end of a primer; the long element is PCR amplified. (C) SFs are synthesized chemically. As extra oligos are not required, the cost of two SFs is not much higher than the total cost of the three elements.

To generate the two-half structure, SFs are usually constructed by joining two DNA elements together using OE-PCR. For example, in **Fig. 1**, *kan* (selectable marker), ori (replication origin) and gene1–4 are first PCR-isolated from their initial sources. Then each two neighboring ones are joined together by OE-PCR to generate SF *kan*-gene1, SF gene1-gene2, SF gene2-gene3, SF gene3-gene4, SF gene4-ori. SF ori-*kan* can be simply PCR-amplified from a vector plasmid and work as a vector backbone. In practice we also successfully got SFs by tailed primer PCR or chemical synthesis (illustrated in **Fig. 2**).

### Combination of SFs by hybridization

**Figure 3:**
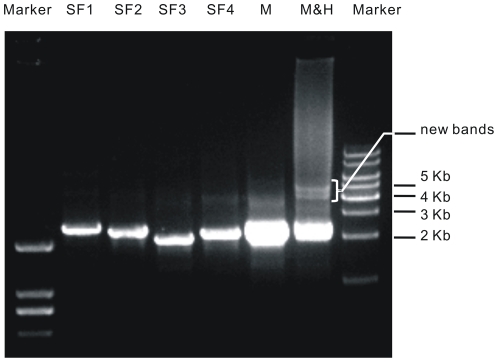
Combination of SFs after renaturation. SF 1–4 (**pOKCA construction,** **Fig 4A** **and Fig. S1**) were mixed (resulting M), denatured and renatured (resulting M&H). They were added to agarose gel in a volume ratio of 1∶1∶1∶1∶4∶4. From the comparison between M and M&H, we can find that, the bands corresponding to SF 1–4 weakened while at least two new bands emerged after the hybridization treatment. The new bands are around 4 and 5 kb, about twice bigger than the four SFs, suggesting that they are resulted from inter-fragment hybridization. M&H was further transformed into *E. coli* DH5α and generated plasmid pOKCA.

The core step of our method, hybridization, was carried out simply with a boiling water bath treatment (see **Materials and Methods**). To see the impact directly, we analyzed the DNAs by gel electrophoresis. As shown in **Fig. 3** after the hybridization treatment, SFs were combined into larger structures.

### Construction of plasmids from four, six and eight SFs

**Figure 4:**
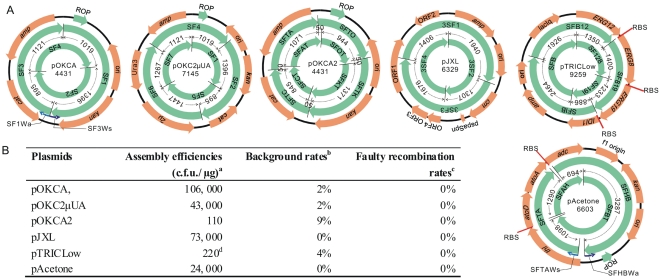
Plasmids constructed in this article and their assembly efficiencies. (**A**) The outer cycle is a plasmid map showing the main genetic elements; two inner green cycles show the overlapped SFs, along with the overlap lengths (bp); the center is plasmid name and size (bp). Two examples are provided to show how nicks or gaps form. In pOKCA construction, the antisense primer for SF1 (SF1Wa) and the sense primer for SF3 (SF3Ws) were designed to be back to back, such that a nick formed between SF1 and SF3. In pAcetone construction, primers SFTAWs and SFHBWa were designed to be apart, so a 110 bp gap formed between SFTA and SFHB. (B) ^a^Colony forming units per microgram DNA. ^b^The proportion of transformants caused by undigested PCR template, determined by colony PCR. ^c^The proportion of unwanted recombinants, determined by restriction mapping (**Fig. S2**) and DNA sequencing. ^d^This plasmid was difficult to construct inherently (see text for details).

As a proof of concept and to evaluate the assembly efficiency, we first constructed three plasmids, pOKCA, pOKC2μUA and pOKCA2 from four, six and eight SFs respectively. Plasmid structures, overlapped SFs and the overlap lengths are illustrated in **Fig. 4A**. The genetic elements assembled were: O, the ori from pET28a (originally from pBR322); K, kanamycin resistance gene *kan*; C, chloramphenicol resistance gene *cat*; A, ampicillin resistance gene *amp*; 2μ, 2μ replication origin; U, yeast *ura3* gene. Most SFs were prepared by jointing two elements together by OE-PCR. SFs containing 50 bp overlaps were prepared by tailed primer PCR (details of SF preparations were illustrated in **Fig. S1**). After mixing, DpnI digestion and hybridization, 5 microliters of SFs were transformed into DH5α chemical competent cells. The results were summarized in **Fig. 4** **B**. As shown, SHA was able to assemble up to eight SFs with overlaps down to 50 bp with sufficient efficiency. In contrast, mixture without hybridization treatment gave no or only a few colonies which were traced to undigested plasmid.

**Figure 5:**
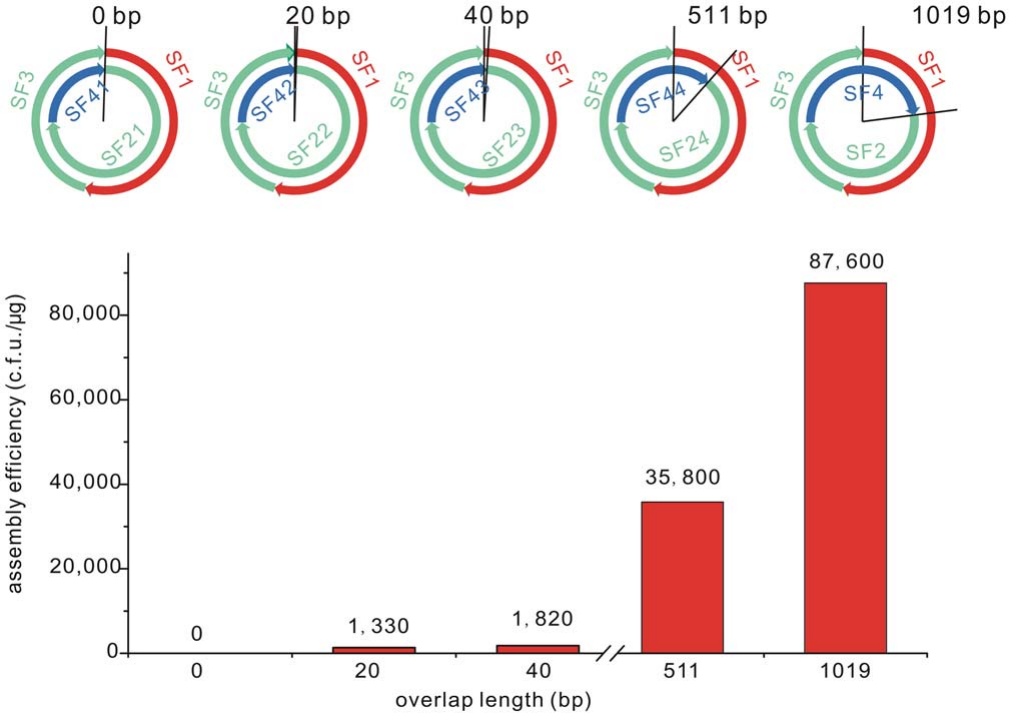
The impact of overlap length on assembly efficiency. Five reconstruction of pOKCA were designed, in which the overlap between SF1 (red) and SF4X (blue, stands for SF4, SF41, SF42, SF43, or SF44) varied from 0 bp to 1019 bp. SF1 and SF3 were prepared as described in the text (and **Fig. S1**). SF4X and SF2X (SF2, SF21, SF22, SF23, or SF24) were amplified from pOKC2μUA with corresponding primers (**Table S2**). Transformations were selected on Cm plates. Assembly efficiencies are given as colony forming units per microgram of SFs.

By varying overlap length we found that the longer overlap used the higher assembly efficiency was achieved, and no overlap led to no assembly (**Fig. 5**).

### Construction of a customized vector

A vector is typically composed of a replication origin, a selectable marker, multiple cloning sites and some other elements which can endow it with different functional traits, such as copy number, maintenance stability, induction method, and expression intensity. Existing vectors usually can't exactly meet all the demands for a specific task. So it is very desirable if we can pick every element we need and assemble them into a vector right before using.

Here we constructed a customized vector, pJXL (**Fig. 4**), for *B. subtilis* metabolic engineering within two days. It was consisted of four DNA pieces: the *Amp* and ori of pBR322, the 3 kb replication region of plasmid pBS72 [4], [5], and the chloramphenicol acetyltransferase gene and *spaS* promoter from plasmid pNZ8901 [6]. Two SFs were chemically synthesized; the other two were prepared by OE-PCR (details were illustrated in **Fig. S1**).

The function of constructed pJXL was demonstrated by controlled production of enhanced green fluorescent protein reporter (**Fig. S3**).

### Construction of a 4-gene partial isoprene-biosynthesis pathway

Previously, a similar pathway was constructed for terpenoids production by two rounds of traditional cloning and OE-PCR [7]. Here using SHA, we finished the construction, named pTRICLow (**Fig. 4A**), within two days, including the time for SFs preparation. Genes encoding the last four enzymes of the MVA pathway, *ERG12*, *ERG8*, *ERG19*, and *IDI1*, were amplified from *Saccharomyces cerevisiae* genomic DNA individually; two vector fragments, B5′ fragment and B3′ fragment, were amplified from expression vector pTrcHis2A. From them six SFs were prepared by OE-PCR. Three RBS-containing sequences of 45 bp each were added to separate and organize all 4 genes into a single synthetic operon (details about SF preparations were illustrated in **Fig. S1**). The assembly efficiency was sufficient but lower than expected (**Fig. 4B**). To investigate the reason, we retransformed DH5α cells with the constructed pTRICLow (in covalently closed circular form). The efficiency was about two orders of magnitude lower than normal plasmids with similar sizes. Thus this plasmid was difficult to construct inherently. Similar phenomenon was also reported for traditional cloning methods [8].

pTRICLow, was then cotransformed with pACY-IspS4 (coding for a modified *Populus alba* isoprene synthase, constructed previously in our lab by traditional cloning, see **Method S1** for details.) into *E. coli* BL21 (DE3). Isoprene production was confirmed by GC-MS. (see **Fig. S3**)

### Construction of a 4-gene acetone-biosynthesis pathway

During the above constructions, we designed the SFs (by choosing the primer positions, examples were shown in **Fig. 4A**) in a way that after the hybridization nicks formed between SFs. It was previously reported that, like nicks, gaps (of 6-nt, in the LIC method) could also be repaired by the cells [9]. Here, we thought our method should be able to tolerate even longer gaps, since it could stick the fragments together more stably (the complementary regions were much longer than in LIC method). The benefit of this ability would be making the primer designing easier and providing more flexibility.

To test it, we constructed a synthetic acetone pathway, pAcetone, with gaps of 110, 54, 58 and 12 nt formed after the hybridization (**Fig. 4A**). This pathway was derived from the isopropanol production pathway first designed by Hanai *et al* [10]. *thl* and *adc* were amplified from *Clostridium acetobutylicum* ATCC 824 genomic DNA individually; *atoA* and *atoD* were amplified from *E. coli* K12 genomic DNA together; the recipient vector was amplified from plasmid pET28aΔ*lacI* which was reduced from pET28a (see **Method S2** for details). Then by OE-PCR, they were joined into 4 SFs. Similar to what we did in pTRICLow construction, two RBS-containing sequences were inserted between the genes (details was illustrated in **Fig. S1**).

In order to evaluate the impact of the long gaps, we reconstructed this plasmid in a no-gap way (**Method S3**). The results demonstrated that our method still held 76% of its efficiency with gaps comparing to the no-gap control.

Then pAcetone was transformed into *E. coli* BL21 (DE3). Acetone production was confirmed by GC-MS (Fig. S3).

### Fidelity of SHA

We sequenced all the constructs, total of 51.9 kb. Two mutations were found to be introduced by the primers used and one mutation was introduced by DNA polymerase during strand extension (see **Table S1** for details). This high fidelity was achieved by applying high-fidelity PCR mix and avoiding gel purification in which DNA might be damaged by the UV light. Hybridization itself isn't likely to generate any mutation.

### Faulty recombination

In contrast to previous methods, no faulty recombination was found for SHA according to the restriction mapping (**Fig. S2**) and DNA sequencing results. This should owe to the special mechanism of SHA, which relies on the recognition between long DNA sequences compared with previous methods relying on the recognition between short sequences (usually no longer than 50 bp).

**Figure 6:**
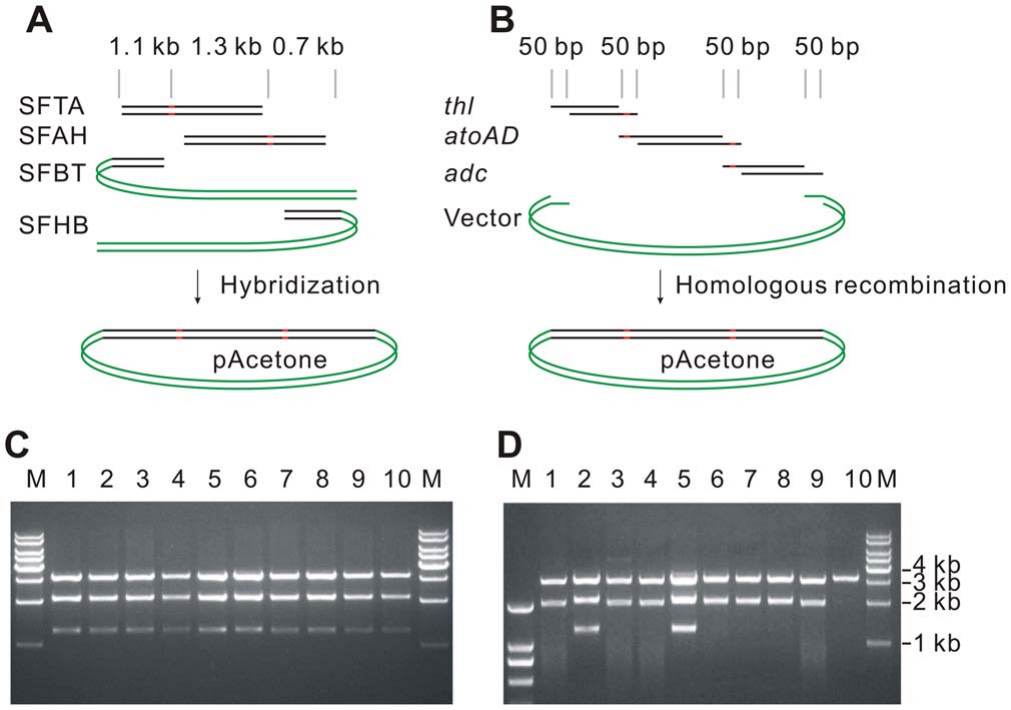
Constructing pAcetone by SHA and SLIC. (**A**) Constructing pAcetone by SHA. SFs recognized each other with complementary areas of 0.7–1.3 kb. (**B**) Constructing pAcetone by SLIC. Fragments recognized each other with their 50 bp homologous ends which were single stranded by T4 DNA polymerase treatment. Repeated sequences (15 bp RBS+3 bp ATG) is highlighted in red. Vector backbone (green) was amplified from pET28aΔlacI. Insert fragments *thl*, *atoAD* and *adc* with homologous ends were generated by PCR (**Table S4**). (**C**) 10 randomly picked constructions from **A** were digested with *Bgl*II and *Sal*I. 100% gave out correct bands (1.2 kb, 2.1 kb, and 3.2 kb). (**D**) 10 randomly picked constructions from **B** were digested with *Bgl*II and *Sal*I. Only two (lanes 2 and 5) had correct bands. After sequencing, faulty recombinations were determined to be lacking of *atoAD* (lanes 1, 3, 4, 6–9) or lacking of all three inserts (lane 10).

To compare them more directly, we attempted to construct the pAcetone using SLIC. This was only a 4-fragment assembly. Every fragment had two 50 bp homologous ends that mediate the construction. RBS together with start codon, total of 18 bp, repeat twice in the homologous areas. In the previous study, SLIC had a faulty recombination rate of 0% for 5-fragment assembly, but 83% for 10-fragment assembly [2]. Here we found that the short 18 bp repeats made the situation even worse. As shown in **Fig. 6**, out of ten random picked transformants only two were assembled correctly. Seven lost *acoAD* insert; one lost all three inserts (it was distinguished from pET28aΔ*lacI* background by that it contained a residual insert corresponding to the first 28 bp of *thl*).

## Discussion

DNA hybridization was traditionally used for southern blotting and DNA microarray. Here, we adapted it for DNA construction, and developed an efficient method, termed SHA. By harnessing the hybridization of SFs which were prepared to overlap each other, SHA can assemble multiple DNA fragments in a one-step fashion within two days. Unlike conventional cloning approaches that use restriction endonucleases or recombinases, SHA is sequence-independent, and doesn't require special equipment or enzyme. Firstly we demonstrated its feasibility by constructing plasmids from 4, 6 and 8 fragments. The assembly efficiency was found to be positively affected by overlap length and negatively affected by SF numbers. Then we showed its practical value in pathway and vector constructions.

SHA depends on the recognition between long DNA sequences. The whole sequence of every DNA fragment takes part in it. In previous methods, only a little part (usually no longer than 50 bp) of the DNA fragment takes part in the recognition. Firstly, this difference makes SHA more robust and efficient. DNAs don't need to be gel purified or modified by any enzymes; much longer gaps can be tolerated. More importantly, unlike previous methods, SHA is not affected by repeat sequences. In synthetic biology, as people commonly need to use a genetic element, such as a promoter or a RBS, more than once for multi-gene expression, SHA would be a better choice.

## Materials and Methods

### Bacterial strains

*E. coli* DH5α was used for SHA and *E. coli* BL21 (DE3) was used for gene expression.

### Media, chemicals and enzymes

We used Luria-Bertani broth for culturing all our strains. Agar (1.5%) was added to make solid medium. Antibiotics were used if necessary as follows: ampicillin (Amp, 50 µg ml^−1^), kanamycin (Kan, 50 µg ml^−1^), and chloramphenicol (Cm, 30 µg ml^−1^ for *E. coli* and 5 µg ml^−1^ for *B. subtilis*). IPTG was used to induce gene expression at 0.5 mM.

Pyrobest™ DNA polymerase from TAKARA was used for SF preparation. EasyTaq™ DNA polymerase from Transgen was used for colony PCR screening.

### SF preparation

OE-PCR was performed by the version of Shevchuk et al. [11], except that Pyrobest™ DNA polymerase was used throughout. OE-ends had lengths of 30–50 bp. By following their two tips we can always obtain high product yield and purity on the first attempt. First, use the two-step model including an overlap extension step without primers (step A) and an exponential amplification step with primers (step B); second, use nested primers, i. e. in step B the primers (called wing primers or flanking primers) should be within the product from step A, and have a distance above 50 bp from the ends. The PCR mixtures (each 50 µl) were made according to manufacturer's instructions: 0.2 mM each of dNTP, 1×Pyrobest Buffer II, 0.025 U µl^−1^ Pyrobest™ polymerase, 0.4 µM each of primers (for step B) or no primers (for step A), and template. For step A, two unpurified PCR-isolated elements were used as template at a final concentration of 5 ng µl^−1^ each; for step B, 2 µl of unpurified product from step A was used as template. Cycling parameters: for step A, initial denaturation 94°C 2 min, subsequent steps 94°C15 s, annealing at 56°C 20 s, extension 72°C 2 min, 10 cycles total, hold at 4°C; for step B, initial denaturation 94°C 2 min, subsequent steps 94°C 15 s, annealing at 62°C 20 s, extension 72°C 4 min, 35 cycles total, final additional extension 72°C 3 min, hold at 4°C. PCR templates and primers are all given in **Table S2**; primer sequences are given in **Table S3**. Plasmid templates were linearized with appropriate restriction enzymes before using (**Table S2**.).

Tailed primer PCR was performed similarly to regular PCR, except that the primer had a 5′ end overhang. In this case (pOKCA2 construction), SFs were purified by E.Z.N.A.™ Cycle-Pure kit (OMEGA) to exclude residual primer oligonucleotides whose lengths were comparable to that of the overlaps. In other cases, purification was omitted.

DNA chemical synthesis was conducted by the Shanghai Generay Biotech Co.

### Successive hybridization

Prepared SFs were checked by agarose gel electrophoresis. Roughly equal molar amounts of SFs (about 200 ng µl^−1^ each) were mixed in a 1.5 ml polyethylene tube, treated with DpnI at 37°C for 3 h to digest the plasmid templates, then sealed with Parafilm for water-proof and submerged in a beaker of boiling water (about 97–100°C), left there, till the water cooled to room temperature (18–25°C) which usually took about 2 hours.

The reaction was transformed into *E. coli* competent cells with standard heat shock method [12].

### Restriction mapping of the constructed plasmids

Plasmids were isolated from overnight cultures using E.Z.N.A. ™ Plasmid Mini Kit from OMEGA, subjected to restriction enzyme digestion, and then loaded to 1% agarose gels to check their restriction digestion patterns by DNA electrophoresis. Restriction enzymes used for every plasmid were listed in **Fig. S2**.

## Supporting Information

Figure S1 **Details of SF preparations.**
(PDF)

Figure S2 **Restriction mapping results.**
(PDF)

Figure S3 **Functional analyses of pJXL, pTRIClow and pAcetone.**
(PDF)

Table S1 **DNA sequencing results.**
(PDF)

Table S2 **PCR templates and primers for SF preparations.**
(PDF)

Table S3 **Primer sequences.**
(PDF)

Table S4 **Construction of pAcetone by SLIC.**
(PDF)

Method S1 **Cloning** ***Populus alba*** **isoprene synthase.**
(PDF)

Method S2 **Construction of pET28aΔ** ***lacI*** **.**
(PDF)

Method S3 **Reconstructing pAcetone in a no-gap way.**
(PDF)
