# Supplementary material for: In Vitro Assembly of Multiple DNA Fragments Using Successive Hybridization
Source: PLoS One. 2012 Jan 26;7(1):e30267. doi: 10.1371/journal.pone.0030267 (PMC3266897; doi:10.1371/journal.pone.0030267)
Supplement: Figure S1 — Details of SF preparations. (PDF) [file pone.0030267.s001.pdf]

# FigureS1 Details of SF preparations

## Nucleic Acids nomenclature：

1. SFs were named as SF[capital letters or numbers] (for example, SF2, SFTO and SF19I) or [a number]SF[capital letters or numbers] (for example, 3SF1, 3SF2, 3SF3, 3SF4 were for constructing pJXL, while SF1, SF2, SF3, SF4 were for constructing pOKCA).
2. In the case that the SF was prepared by OE-PCR from two DNA fragments, those two DNA fragments were named as [the name of the SF]h[capital letters or numbers]. For example, SF2 was prepared from SF2hK and SF2hC; SF19I was prepared from SF19Ih19 and SF19IhI.
3. The sense primer used to amplify a SF was named as [the name of the SF]Ws; the antisense primer used to amplify the SF was named as [the name of the SF]Wa ( The PCR template can be a plasmid in a regular PCR or two DNA fragments in an OE-PCR). For example, SF1 was amplified with SF1Ws and SF1Wa (from pET28a); SF2 was amplified with SF2Ws and SF2Wa (from SF2hK and SF2hC).
4. For amplifying the DNA fragments (used as templates in the OE-PCR, see above **2**), the primers were named as [the name of the DNA fragment]s and [the name of the DNA fragment]a for sense primers and antisense primers respectively. For example, SF2hK was amplified with SF2hKs and SF2hKa.

**Details of the constructions of pOKCA and pOKC2μUA.**

**(A)** SF preparations. The preparations of SF1 and SF2 are given as examples. Others can be found in **Table S2**. SF1 was directly PCR-amplified from pET28a, because the ori and *kan* were already together there. SF2 was prepared by OE-PCR. A 25 bp segment of pACYduet1 was added to the 5' end of primer SF2hKa. Likewise a 25 bp segment of pET28a was added to the 5' end of primer SF2hCs. This causes the two PCR products, SF2hK and SF2hC to have an overlap region of 50 bp which mediate the OE-PCR that generated SF2. pET28a and pACYduet1 were linearized by HindIII before use.

**(B)** Constructing by SHA. SF 1-4 were hybridized to get pOKCA and SF 1,2,5,6,7,4 were hybridized to get pOKC2μUA. Overlaps between SFs and their lengths are given. Hybridization was conducted as described in **section Materials and Methods.**

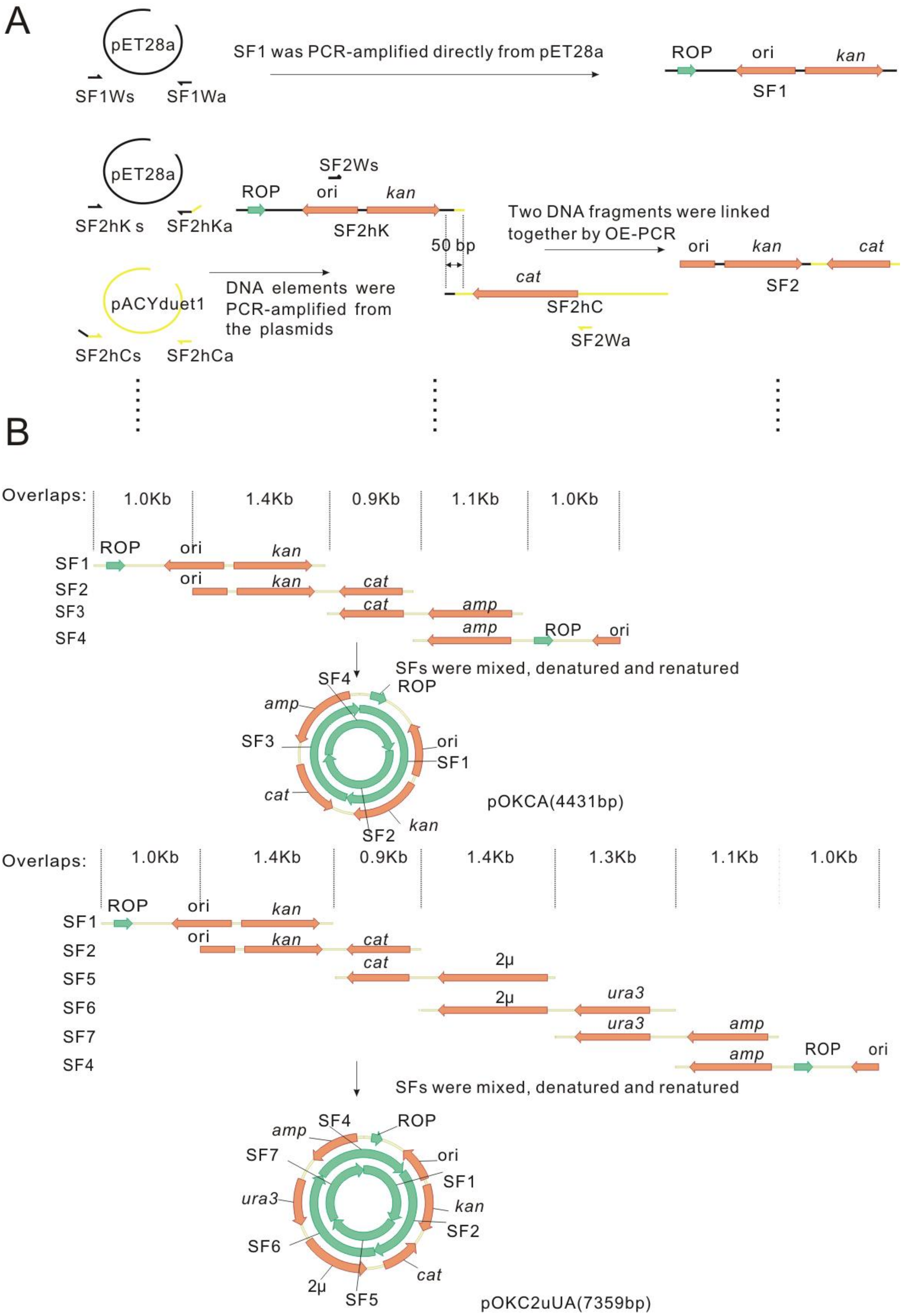
A
pET28a
SF1Ws
SF1Wa
SF1 was PCR-amplified directly from pET28a
ROP
ori
kan
SF1
pET28a
SF2hK s
SF2hKa
SF2Ws
ROP
ori
kan
SF2hK
50 bp
Two DNA fragments were linked together by OE-PCR
ori
kan
cat
SF2
pACYduet1
SF2hCs
SF2hCa
DNA elements were PCR-amplified from the plasmids
cat
SF2hC
SF2Wa
B
Overlaps:
1.0Kb
1.4Kb
0.9Kb
1.1Kb
1.0Kb
SF1
SF2
SF3
SF4
ROP
ori
kan
cat
amp
SFs were mixed, denatured and renatured
pOKCA(4431bp)
Overlaps:
1.0Kb
1.4Kb
0.9Kb
1.4Kb
1.3Kb
1.1Kb
1.0Kb
SF1
SF2
SF5
SF6
SF7
SF4
2μ
ura3
amp
ROP
ori
SFs were mixed, denatured and renatured
pOKC2uUA(7359bp)

**Details of the construction of pOKCA2.**

**(A)** SF preparations. The preparations of SFTK, SFKT, SFTC and SFCT are given as examples. 25 bp from pACYduet1 was included in primer SFKTa; 25 bp from pET28a was included in primer SFTCs. As a result, SFKT and SFTC overlap with each other for 50 bp. Preparations of the other SFs can be found in **Table S2**. pET28a and pACYduet1 were linearized by HindIII before use.

**(B)** Constructing by SHA. Eight SFs were hybridized to get pOKCA2. Overlaps between SFs and their lengths are given. Hybridization was conducted as described in **section Materials and Methods.**

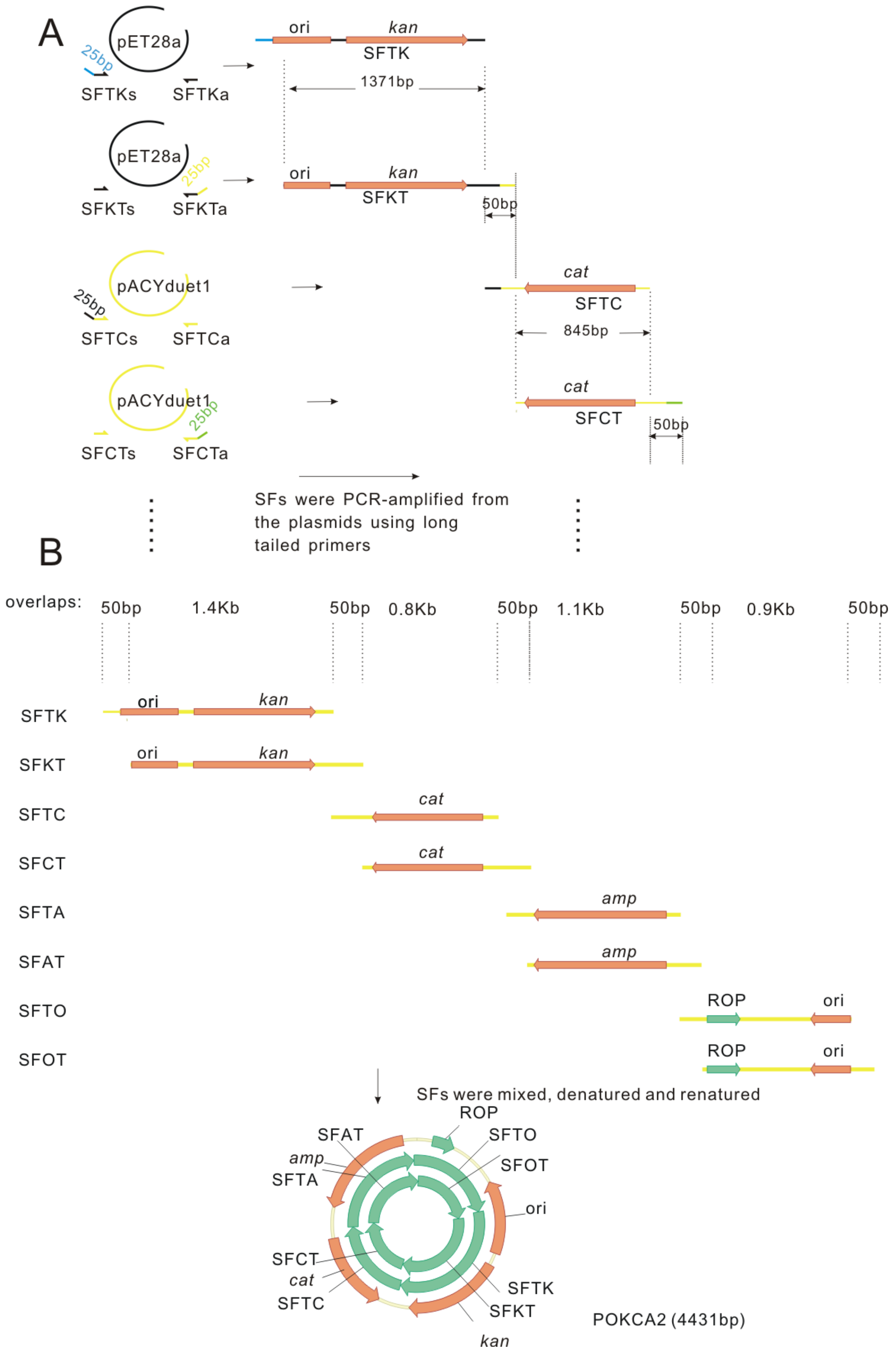

A
pET28a
25bp
SFTKs
SFTKa
ori
kan
SFTK
1371bp
SFKTs
SFKTa
SFKT
50bp
pACYduet1
SFTCs
SFTCa
cat
SFTC
845bp
SFCTs
SFCTa
SFCT
SFs were PCR-amplified from the plasmids using long tailed primers
B
overlaps:
50bp
1.4Kb
0.8Kb
1.1Kb
0.9Kb
SFTA
SFAT
amp
SFTO
SFOT
ROP
SFs were mixed, denatured and renatured
POKCA2 (4431bp)

**Details of the construction of pJXL.**

**(A)** SF preparations. The preparations of all SFs are given. They can also be found in **Table S2**. pBR322 were linearized by BamHI before use. 3SF3 was chemically synthesized along with a 50 bp sequence from pBR322 which was subsequently used to immediate the OE-PCR to generate 3SF1. Similarly, 3SF4 was chemically synthesized along with another 50 bp sequence from pBR322 which was subsequently used to immediate the OE-PCR to generate 3SF2.

**(B)** Constructing by SHA. Overlaps between SFs and their lengths are given. Hybridization was conducted as described in **section Successive hybridization Materials and Methods**

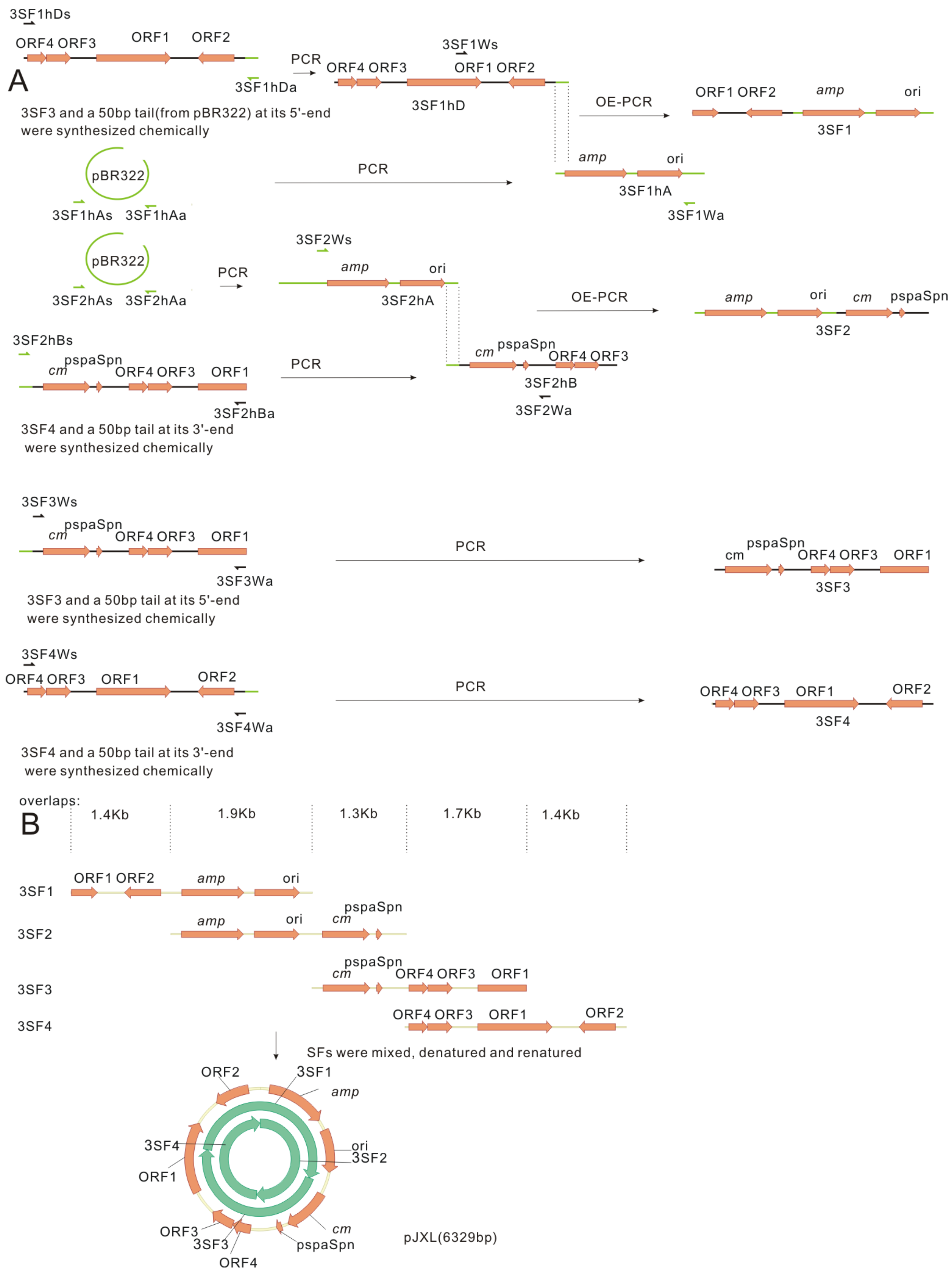
A
3SF1hDs
ORF4 ORF3
ORF1
ORF2
3SF1hDa
3SF3 and a 50bp tail(from pBR322) at its 5'-end were synthesized chemically
PCR
3SF1Ws
ORF4 ORF3
ORF1 ORF2
3SF1hD
OE-PCR
ORF1 ORF2
amp
ori
3SF1
pBR322
3SF1hAs
3SF1hAa
PCR
amp
ori
3SF1hA
3SF1Wa
pBR322
3SF2hAs
3SF2hAa
PCR
3SF2Ws
amp
ori
3SF2hA
OE-PCR
amp
ori
cm
pspaSpn
3SF2
3SF2hBs
cm
pspaSpn
ORF4 ORF3
ORF1
3SF2hBa
3SF4 and a 50bp tail at its 3'-end were synthesized chemically
PCR
cm
pspaSpn
ORF4 ORF3
3SF2hB
3SF2Wa
3SF3Ws
cm
pspaSpn
ORF4 ORF3
ORF1
3SF3Wa
3SF3 and a 50bp tail at its 5'-end were synthesized chemically
PCR
cm
pspaSpn
ORF4 ORF3
ORF1
3SF3
3SF4Ws
ORF4 ORF3
ORF1
ORF2
3SF4Wa
3SF4 and a 50bp tail at its 3'-end were synthesized chemically
PCR
ORF4 ORF3
ORF1
ORF2
3SF4
B
overlaps:
1.4Kb
1.9Kb
1.3Kb
1.7Kb
1.4Kb
3SF1
ORF1 ORF2
amp
ori
3SF2
amp
ori
cm
pspaSpn
3SF3
cm
pspaSpn
ORF4 ORF3
ORF1
3SF4
ORF4 ORF3
ORF1
ORF2
SFs were mixed, denatured and renatured
ORF2
3SF1
amp
3SF4
ORF1
ori
3SF2
ORF3
3SF3
ORF4
cm
pspaSpn
pJXL(6329bp)

**Details of the construction of pTRICLow.**

**(A)** SF preparations. The preparations of SFB, SFB12, SF128 and SF819 are given as examples. Others can be found in **Table S2**. SFB was amplified directly from pTrcHis2a. SFB12, SF128 and SF819 were prepared by OE-PCR. The RBS sequence is highlighted in red. To place it between ERG12 and ERG8, we included it in primers SF128h12a, SF128h8s, and SF819Ws. pTrcHis2a was linearized by BamHI before use.

**(B)** Constructing by SHA. Overlaps between SFs and their lengths are given. Hybridization was conducted as described in **section Materials and Methods.** When doing this construction, we didn't know it can tolerate gaps yet. So the primer SF819Ws was designed to contain a RBS tail to avoid gap, which was proved to be unnecessary later in the construction of pAcetone**.**

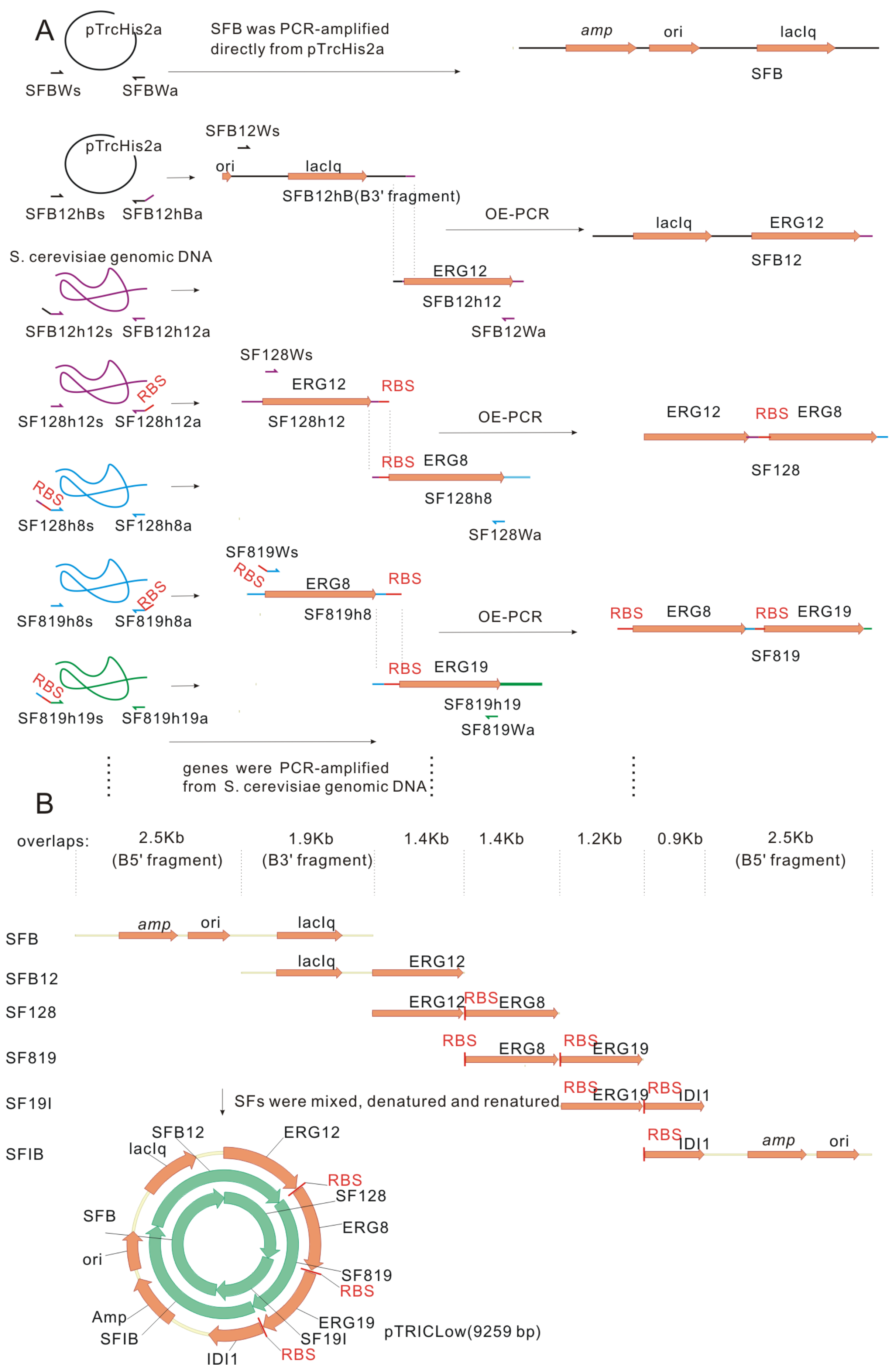
A
pTrcHis2a
SFBWs
SFBWa
SFB was PCR-amplified
directly from pTrcHis2a
amp
ori
lacIq
SFB
SFB12Ws
ori
lacIq
SFB12hBs
SFB12hBa
SFB12hB(B3' fragment)
OE-PCR
lacIq
ERG12
SFB12
S. cerevisiae genomic DNA
ERG12
SFB12h12
SFB12h12s
SFB12h12a
SFB12Wa
SF128Ws
ERG12
RBS
SF128h12
SF128h12s
SF128h12a
OE-PCR
ERG12
RBS
ERG8
SF128
RBS
ERG8
SF128h8
SF128h8s
SF128h8a
SF128Wa
SF819Ws
ERG8
RBS
SF819h8
SF819h8s
SF819h8a
OE-PCR
RBS
ERG8
RBS
ERG19
SF819
RBS
ERG19
SF819h19
SF819h19s
SF819h19a
SF819Wa
genes were PCR-amplified
from S. cerevisiae genomic DNA
B
overlaps:
2.5Kb
(B5' fragment)
1.9Kb
(B3' fragment)
1.4Kb
1.4Kb
1.2Kb
0.9Kb
2.5Kb
(B5' fragment)
SFB
SFB12
SF128
SF819
SF19I
SFIB
amp
ori
lacIq
ERG12
ERG12
RBS
ERG8
RBS
ERG8
RBS
ERG19
RBS
ERG19
RBS
IDI1
RBS
IDI1
amp
ori
SFs were mixed, denatured and renatured
SFB12
lacIq
ERG12
RBS
SF128
ERG8
SFB
ori
SF819
RBS
ERG19
SF19I
Amp
SFIB
IDI1
RBS
pTRICLow(9259 bp)

**Details of the construction of pAcetone.**

**(A)** SF preparations. The preparations of SFBT and SFTA are given as examples. Others can be also found in **Table S2**. pET28aΔ*lacI* （**Text S2**） was linearized by HindIII before use. The RBS sequence is highlighted in red. To place it between *thl* and *atoD*, we included it in primers SFTAhTa and SFTAhAs.

**(B)** Constructing by SHA. Overlaps between SFs and their lengths are given. Gaps of 110, 54, 58 and 12 nt existed in the hybridization complex (see text for detail). Hybridization was conducted as described in **section Materials and Methods**

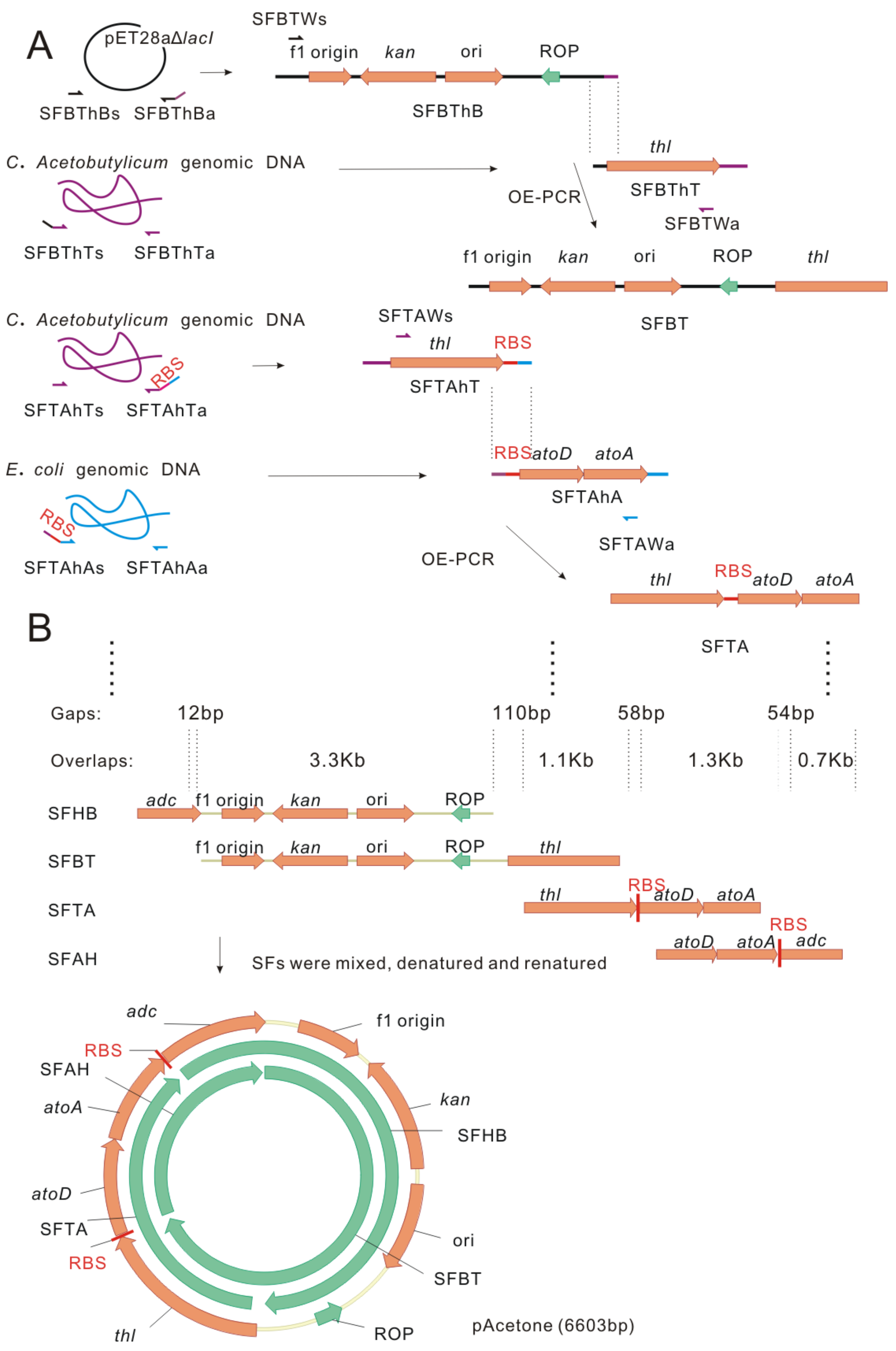
A
pET28aΔlacI
SFBThBs SFBThBa
SFBTWs
f1 origin kan ori ROP
SFBThB
C. Acetobutylicum genomic DNA
SFBThTs SFBThTa
thl
SFBThT
OE-PCR
SFBTWa
f1 origin kan ori ROP thl
SFBT
C. Acetobutylicum genomic DNA
RBS
SFTAhTs SFTAhTa
SFTAWs
thl RBS
SFTAhT
E. coli genomic DNA
RBS
SFTAhAs SFTAhAa
RBS atoD atoA
SFTAhA
SFTAWa
OE-PCR
thl RBS atoD atoA
SFTA
B
Gaps: 12bp 110bp 58bp 54bp
Overlaps: 3.3Kb 1.1Kb 1.3Kb 0.7Kb
SFHB adc f1 origin kan ori ROP
SFBT f1 origin kan ori ROP thl
SFTA thl RBS atoD atoA
SFAH atoD atoA RBS adc
SFs were mixed, denatured and renatured
adc
f1 origin
RBS
SFAH
kan
atoA
SFHB
atoD
SFTA
RBS
ori
SFBT
thl
ROP
pAcetone (6603bp)
