# Supplementary material for: In Vitro Assembly of Multiple DNA Fragments Using Successive Hybridization
Source: PLoS One. 2012 Jan 26;7(1):e30267. doi: 10.1371/journal.pone.0030267 (PMC3266897; doi:10.1371/journal.pone.0030267)

Figure S2 Restriction mapping results.

(A) pOKCA (B) pOKC2μUA (C) pOKCA2 (D) pTRIClow (E) pJXL (F) pAcetone.

Restriction endonucleases used and the expected results are as follows:

plasmids	Restriction endonucleases	Fragments generated
**pOKCA**	ApalI	500bp+737bp+3194bp
**pOKC2μUA**	NcoI, AvaI	1759bp+2036bp+3350bp
**pOKCA2**	ApalI	500bp+737bp+3194bp
**pTRIClow**	HindIII, BamHI	1959bp+1098bp+1546bp+4656bp
**pJXL**	ApalI, EcoRI	2281bp+2802bp+1246bp
**pAcetone**	BglII, SalI	1260bp+ 2142bp+3201bp

A

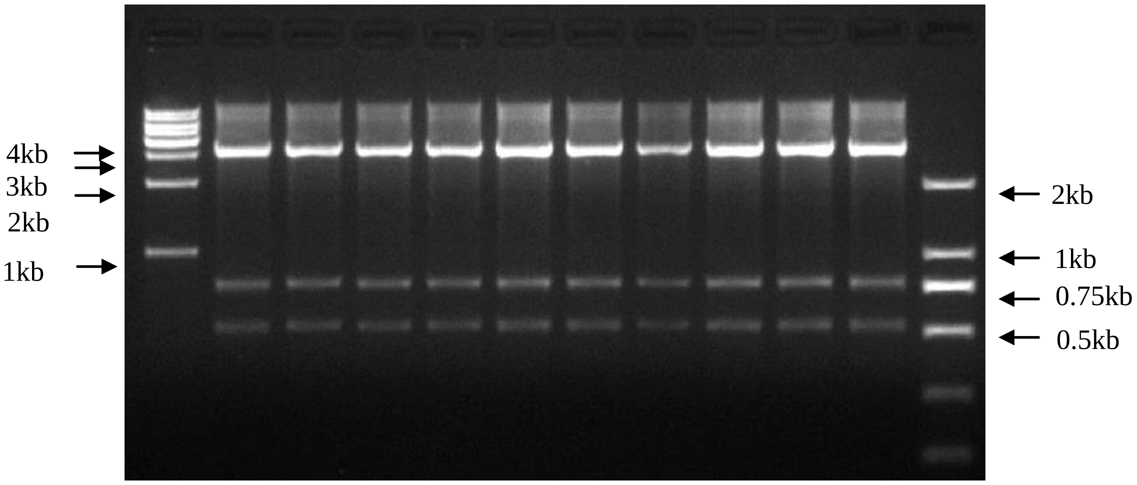

B

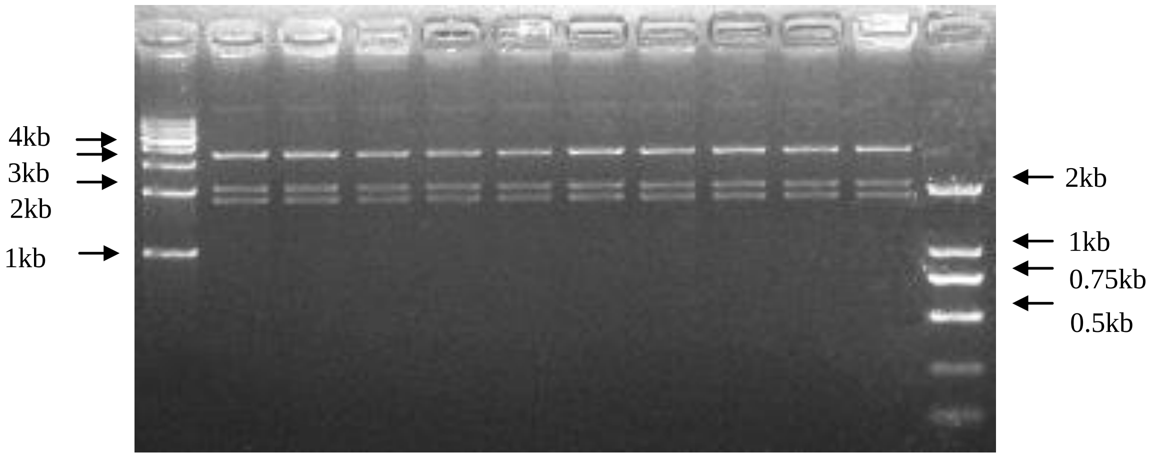
4kb
3kb
2kb
1kb
2kb
1kb
0.75kb
0.5kb

C

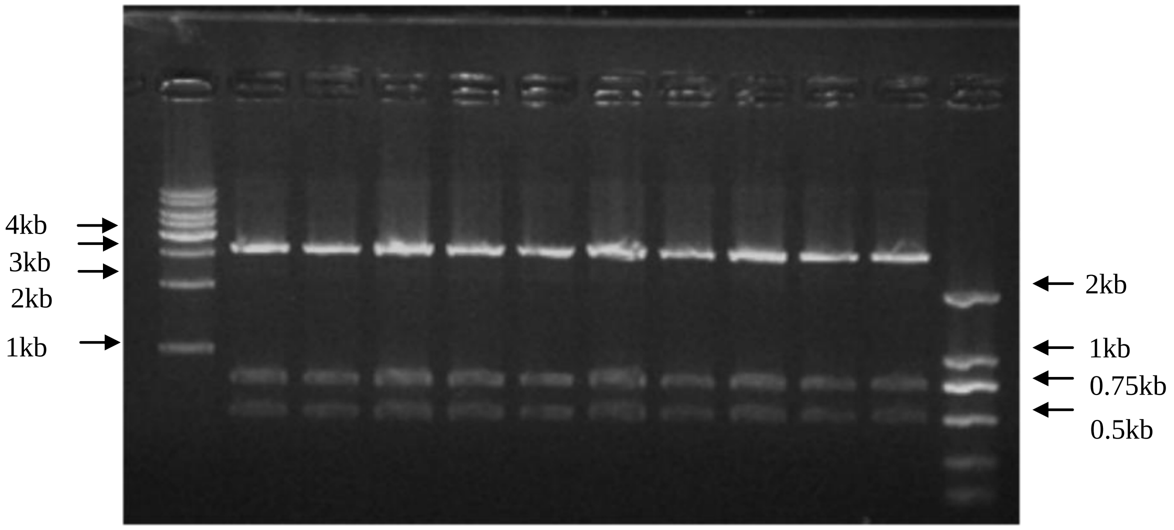
4kb
3kb
2kb
1kb
2kb
1kb
0.75kb
0.5kb

D

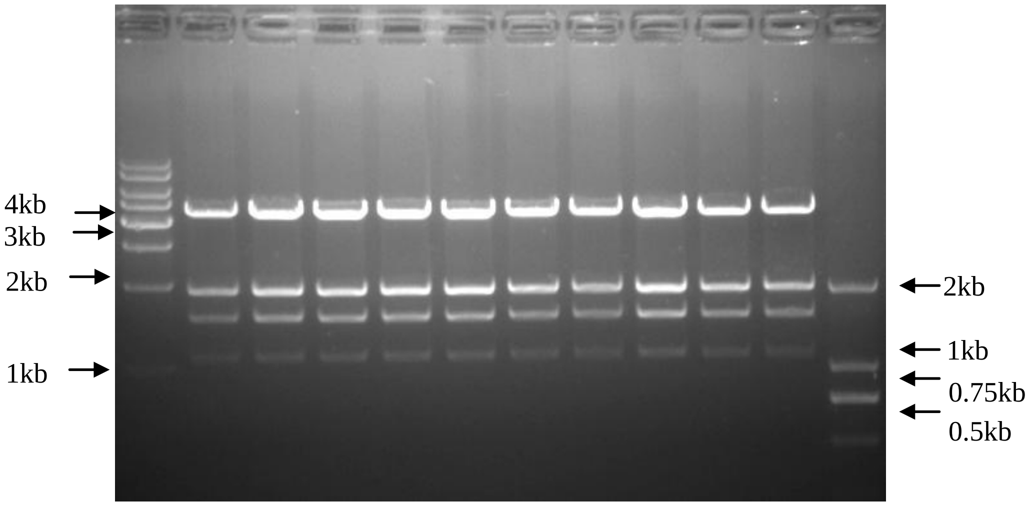
4kb
3kb
2kb
1kb
2kb
1kb
0.75kb
0.5kb

E

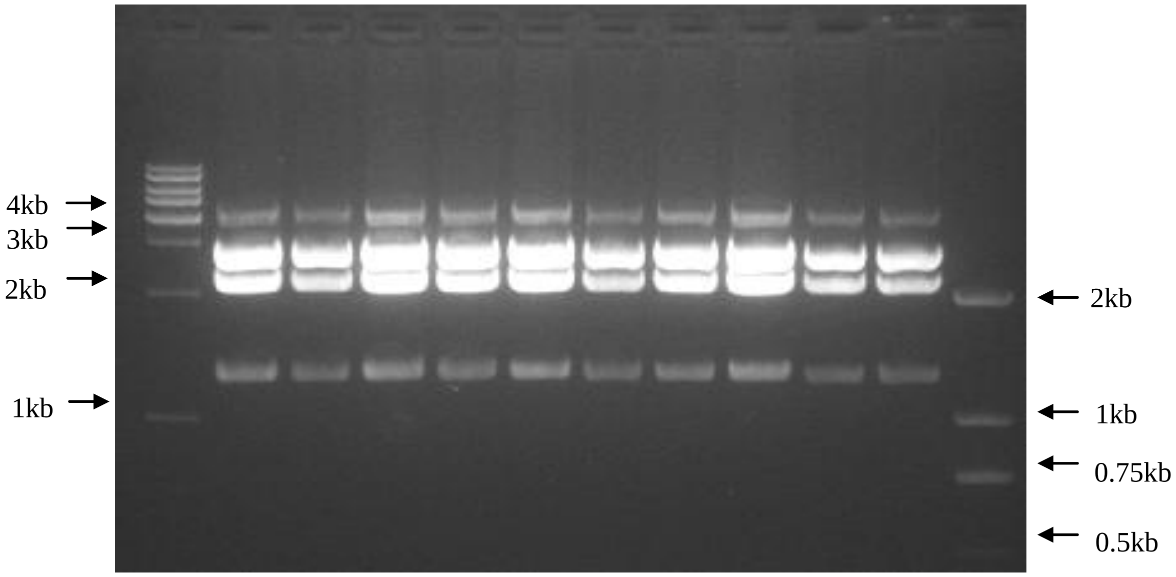

F

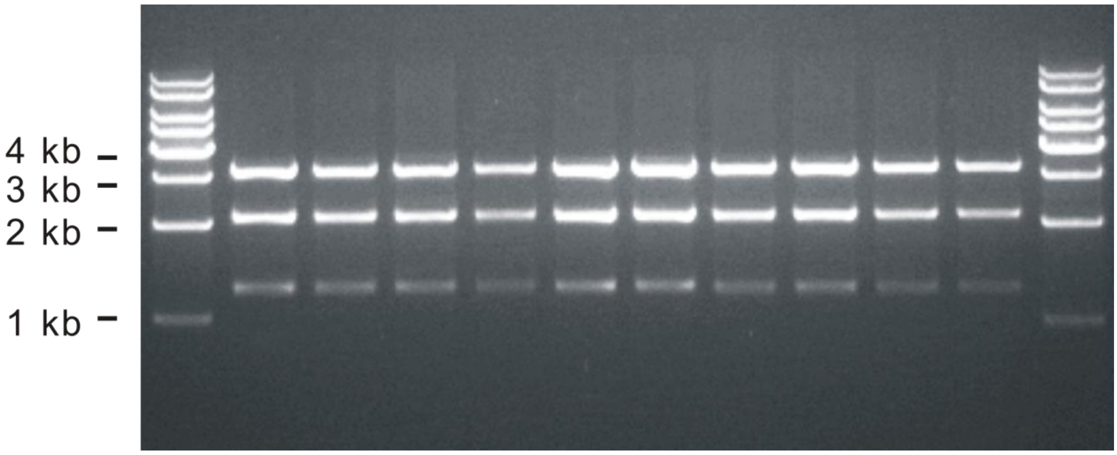

Supplement: Figure S2 — Restriction mapping results. (PDF) [file pone.0030267.s002.pdf]
