# Supplementary material for: In Vitro Assembly of Multiple DNA Fragments Using Successive Hybridization
Source: PLoS One. 2012 Jan 26;7(1):e30267. doi: 10.1371/journal.pone.0030267 (PMC3266897; doi:10.1371/journal.pone.0030267)
Supplement: Figure S3 — Functional analyses of pJXL, pTRIClow and pAcetone. (PDF) [file pone.0030267.s003.pdf]

Figure S3. Functional analyses of pJXL, pTRIClow and pAcetone

Functional analysis of pJXL.

eGFP gene was clone into pJXL by traditional cloning (see below). The resulted pJXL-eGFP was transformed into *B. subtilis* ATCC 6633 by hypertonic electroporation and selected on Cm plate. *B. subtilis* ATCC 6633/pJXL was likewise constructed as negative control. Colonies were picked into LB containing Cm and incubated at 37°C. When the cultures grew to an OD600 of 1.0, they were induced by adding 10% (v/v) subtilin-containing overnight culture supernatant as described previously [1]. After other two hours cells were harvested by centrifugation (10 min, 5,000 g, 4 ℃), washed, resuspended in water and adjusted to an OD600 of 0.5. The fluorescence intensity was measured by a Hitachi F-4600 Fluorescence spectrophotometer and a 1 cm × 1cm quartz cuvette (excitation 488 nm, emission 509 nm). Lane 1：*B. subtilis* ATCC 6633/pJXL not induced; lane 2 *B. subtilis* ATCC 6633/pJXL-eGFP not induced; lane 3：*B. subtilis* ATCC 6633/pJXL induced; lane 2 *B. subtilis* ATCC 6633/pJXL-eGFP induced.

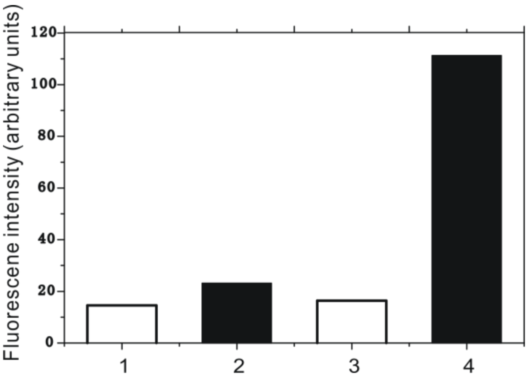

Cloning eGFP gene into pJXL

In our lab the eGFP gene was synthesized chemically and conserved in plasmid pACYduet1(Novagen). The sequence of pACYduet1-GFP is as follows:
catggatatggtgagcaagggcgaggagctgttcaccggggtggtgcccatcctggtcgagctggacggcgacgtaaacggccacaagtt
cagcgtgtccggcgagggcgagggcgatgccacctacggcaagctgaccctgaagttcatctgcaccaccggcaagctgcccgtgccctg

gcccaccctcgtgaccaccctgacctacggcgtgcagtgcttcagccgctaccccgaccacatgaagcagcacgacttcttcaagtccgcca
tgcccgaaggctacgtccaggagcgcaccatcttcttcaaggacgacggcaactacaagacccgcgccgaggtgaagttcgagggcgaca
ccctggtgaaccgcatcgagctgaagggcatcgacttcaaggaggacggcaacatcctggggcacaagctggagtacaactacaacagcc
acaacgtctatatcatggccgacaagcagaagaacggcatcaaggtgaacttcaagatccgccacaacatcgaggacggcagcgtgcagct
cgccgaccactaccagcagaacacccccatcggcgacggccccgtgctgctgcccgacaaccactacctgagcacccagtccgccctga
gcaaagaccccaacgagaagcgcgatcacatggtcctgctggagttcgtgaccgccgccgggatcactctcggcatggacgagctgtaca
agtaagtcgacaagcttgcggccgcataatgcttaagtcgaacagaaagtaatcgtattgtacacggccgcataatcgaaattaatacgactca
ctataggggaattgtgagcggataacaattccccatcttagtatattagttaagtataagaaggagatatacatatggcagatctcaattggatatc
ggccggccacgcgatcgctgacgtcggtacc[KpnI]ctcgagtctggtaaagaaaccgctgctgcgaaatttgaacgccagcacatggactcg
tctactagcgcagcttaattaacctaggctgctgccaccgctgagcaataactagcataaccccttggggcctctaaacgggtcttgaggggttt
tttgctgaaacctcaggcatttgagaagcacacggtcacactgcttccggtagtcaataaaccggtaaaccagcaatagacataagcggctatt
taacgaccctgccctgaaccgacgaccgggtcgaatttgctttcgaatttctgccattcatccgcttattatcacttattcaggcgtagcaccagg
cgtttaagggcaccaataactgccttaaaaaaattacgccccgccctgccactcatcgcagtactgttgtaattcattaagcattctgccgacatg
gaagccatcacagacggcatgatgaacctgaatcgccagcggcatcagcaccttgtcgccttgcgtataatatttgcccatagtgaaaacggg
ggcgaagaagttgtccatattggccacgtttaaatcaaaactggtgaaactcacccagggattggctgagacgaaaaacatattctcaataaac
cctttagggaaataggccaggttttcaccgtaacacgccacatcttgcgaatatatgtgtagaaactgccggaaatcgtcgtggtattcactcca
gagcgatgaaaacgtttcagtttgctcatggaaaacggtgtaacaagggtgaacactatcccatatcaccagctcaccgtctttcattgccatac
ggaactccggatgagcattcatcaggcgggcaagaatgtgaataaaggccggataaaacttgtgcttatttttctttacggtctttaaaaaggcc
gtaatatccagctgaacggtctggttataggtacattgagcaactgactgaaatgcctcaaaatgttctttacgatgccattgggatatatcaacg
gtggtatatccagtgatttttttctccattttagcttccttagctcctgaaaatctcgataactcaaaaaatacgcccggtagtgatcttatttcattatg
gtgaaagttggaacctcttacgtgccgatcaacgtctcattttcgccaaaagttggcccagggcttcccggtatcaacagggacaccaggattt
atttattctgcgaagtgatcttccgtcacaggtatttattcggcgcaaagtgcgtcgggtgatgctgccaacttactgatttagtgtatgatggtgttt
ttgaggtgctccagtggcttctgtttctatcagctgtccctcctgttcagctactgacggggtggtgcgtaacggcaaaagcaccgccggacatc
agcgctagcggagtgtatactggcttactatgttggcactgatgagggtgtcagtgaagtgcttcatgtggcaggagaaaaaaggctgcaccg
gtgcgtcagcagaatatgtgatacaggatatattccgcttcctcgctcactgactcgctacgctcggtcgttcgactgcggcgagcggaaatgg
cttacgaacggggcggagatttcctggaagatgccaggaagatacttaacagggaagtgagagggccgcggcaaagccgtttttccatagg
ctccgcccccctgacaagcatcacgaaatctgacgctcaaatcagtggtggcgaaacccgacaggactataaagataccaggcgtttcccct
ggcggctccctcgtgcgctctcctgttcctgcctttcggtttaccggtgtcattccgctgttatggccgcgtttgtctcattccacgcctgacactca
gttccgggtaggcagttcgctccaagctggactgtatgcacgaaccccccgttcagtccgaccgctgcgccttatccggtaactatcgtcttga
gtccaacccggaaagacatgcaaaagcaccactggcagcagccactggtaattgatttagaggagttagtcttgaagtcatgcgccggttaag
gctaaactgaaaggacaagttttggtgactgcgctcctccaagccagttacctcggttcaaagagttggtagctcagagaaccttcgaaaaacc
gccctgcaaggcggttttttcgttttcagagcaagagattacgcgcagaccaaaacgatctcaagaagatcatcttattaatcagataaaatattt
ctagatttcagtgcaatttatctcttcaaatgtagcacctgaagtcagccccatacgatataagttgtaattctcatgttagtcatgccccgcgccca
ccggaaggagctgactgggttgaaggctctcaagggcatcggtcgagatcccggtgcctaatgagtgagctaacttacattaattgcgttgcg
ctcactgcccgctttccagtcgggaaacctgtcgtgccagctgcattaatgaatcggccaacgcgcggggagaggcggtttgcgtattgggc
gccagggtggtttttcttttcaccagtgagacgggcaacagctgattgcccttcaccgcctggccctgagagagttgcagcaagcggtccacg
ctggtttgccccagcaggcgaaaatcctgtttgatggtggttaacggcgggatataacatgagctgtcttcggtatcgtcgtatcccactaccga
gatgtccgcaccaacgcgcagcccggactcggtaatggcgcgcattgcgcccagcgccatctgatcgttggcaaccagcatcgcagtggg
aacgatgccctcattcagcatttgcatggtttgttgaaaaccggacatggcactccagtcgccttcccgttccgctatcggctgaatttgattgcg
agtgagatatttatgccagccagccagacgcagacgcgccgagacagaacttaatgggcccgctaacagcgcgatttgctggtgacccaat
gcgaccagatgctccacgcccagtcgcgtaccgtcttcatgggagaaaataatactgttgatgggtgtctggtcagagacatcaagaaataac
gccggaacattagtgcaggcagcttccacagcaatggcatcctggtcatccagcggatagttaatgatcagcccactgacgcgttgcgcgag
aagattgtgcaccgccgctttacaggcttcgacgccgcttcgttctaccatcgacaccaccacgctggcacccagttgatcggcgcgagattta
atcgccgcgacaatttgcgacggcgcgtgcagggccagactggaggtggcaacgccaatcagcaacgactgtttgcccgccagttgttgtg

ccacgcggttgggaatgtaattcagctccgccatcgccgcttccactttttcccgcgttttcgcagaaacgtggctggcctggttcaccacgcg ggaaacggtctgataagagacaccggcatactctgcgacatcgtataacgttactggtttcacattcaccaccctgaattgactctcttccgggc gctatcatgccataccgcgaaaggttttgcgccattcgatggtgtccgggatctcgacgctctcccttatgcgactcctgcattaggaaattaata cgactcactataggggaattgtgagcggataacaattcccctgtagaaataattttgtttaactttaataaggagatatac (GFP CDS was highlighted in green)

This template was amplified using primers GFPs (5’-AAAACTGCAG[PstI]TGGATATGGTGAGCAAGGG-3’) and T7ter (5’-TGCTAGTTATTGCTCAGCGG -3’). The PCR product and pJXL were digested with PstI and KpnI and subsequently ligated together. Ligation mixtures were transformed into E. coli DH5a chemical component cells, and the resulting transformants were plated on LB agar with Amp.

Functional analysis of pTRIClow.

Mevalonate (MVA) was prepared by the method of Campos[2] from mevanolactone (Sigma)

pTRICLow, was cotransformed with pACY-IspS4 (coding for a modified *Populus alba* isoprene synthase, constructed previously in our lab by traditional cloning, see **Method S1** for details.) into *E. coli* BL21 (DE3). Colonies were picked into 15 ml LB liquid medium containing Amp and Cm, grown overnight. 0.5 ml fresh overnight cultures were diluted 100-fold and incubated at 37°C to an OD_{600} between 0.6 and 0.8, then treated with 0.5 mM IPTG for 2 hours at 30°C to express the genes. The cultures together with 6 mM MVA were transferred into sterile McCartney bottles and incubated for another 12 h at 30°C. The rubber seals of the McCartney bottles were smeared with silicone grease so that they would not contact with nor absorb the isoprene generated inside the bottle [3]. Both the aerobic and anaerobic incubation were done at 180 rpm on an orbital shaker. For sampling the headspace, an 85 μm polyacrylate fiber was inserted into the McCartney bottle with the help of a manual holder system (Supelco Inc, USA), allowed to equilibrate with the headspace volatiles for 30 min at 50°C and then placed into the GC injector immediately.

GC-MS analyses were performed with an Agilent 5975C GC-MS System equipped with a DB-5ms column (50 m length, 0.25 mm ID, 0.25 μm film thickness, Agilent, Palo Alto, CA, USA). Experimental chromatographic conditions were as follows: injector set at 250°C; Helium carrier gas at 1 ml min^{-1}; oven temperature program: 1 min isotherm at 40°C followed by a linear temperature increase of 5°C

min^{-1} up to 80°C and then 25°C min^{-1} to 300°C held for 5min. MS scan conditions: source temperature 230°C, interface temperature 300°C, E energy 70 eV, mass scan range 2–150 amu. The isoprene peak was identified by both retention time and mass spectrometry

The same strain not treated with MVA was used as control (negative control 2). Strain containing pTrcHis2A (empty plasmid) and pACY-IspS4 treated with MVA was negative control 1. As shown the strain harboring the partial isoprene-biosynthesis pathway gave out a prominent isoprene peak when MVA was added. As *E. coli* can also produce low concentration of dimethylallylpyrophosphate, the substrate for isoprene synthase, by native DXP pathway, a much smaller peak was also observed for negative control 1.

Total ion chromatogram:

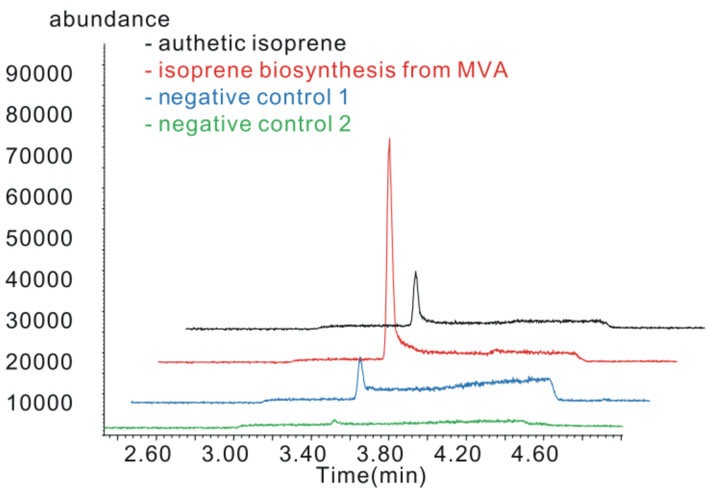

Mass spectra:

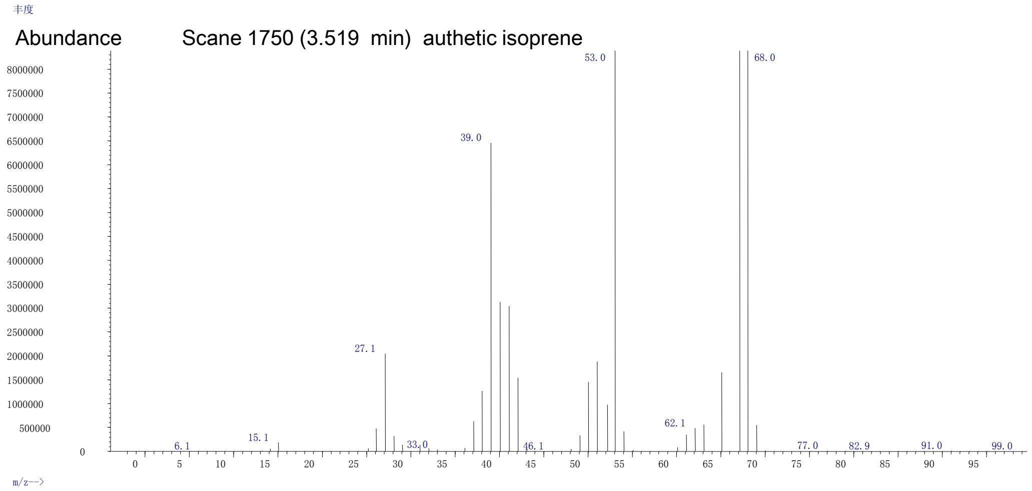
丰度
Abundance
Scane 1750 (3.519 min) authetic isoprene
53.0
68.0
39.0
27.1
62.1
6.1
15.1
33.0
46.1
77.0
82.9
91.0
99.0
m/z-->

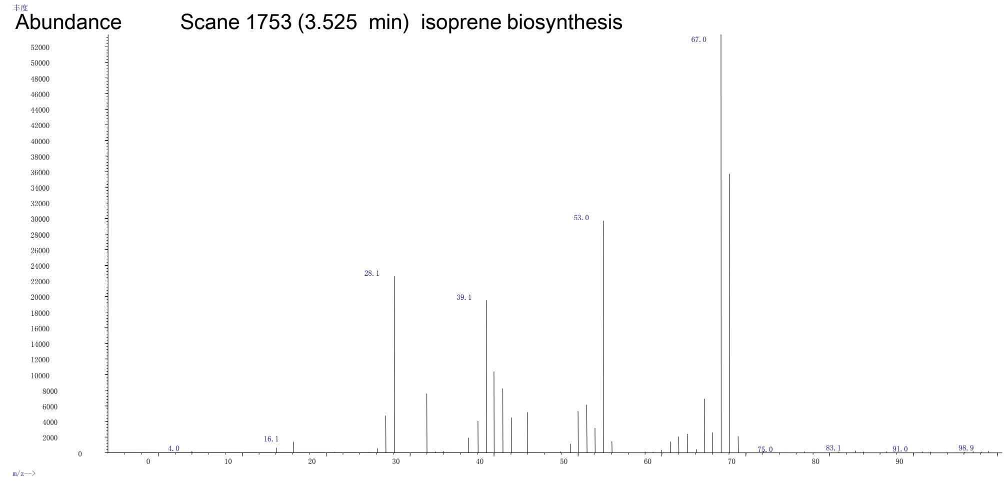
丰度
Abundance
Scane 1753 (3.525 min) isoprene biosynthesis
67.0
53.0
28.1
39.1
4.0
16.1
75.0
83.1
91.0
98.9
m/z-->

Functional analysis of pAcetone.

pAcetone was transformed into *E. coli* BL21(DE3). Colonies were picked into 15 ml LB liquid medium containing Kan, grown overnight, diluted 100-fold and incubated at 37°C to an OD_{600} between 0.6 and 0.8, then added with 0.5 mM IPTG and 2% glucose (w/v), incubated at 30°C for other 12 hours. *E. coli* BL21(DE3) harboring pET28a (the empty vector) was used as negative control. 1μl filtered culture supernatant was analyzed by GC-MS with an Agilent 5975C GC-MS System equipped with a HP-Innowax column (30 m length, 0.25 mm ID, 0.25 μm film thickness, Agilent, Palo Alto, CA, USA). Experimental chromatographic conditions were as follows: injector set at 250°C; Helium carrier gas at 1 ml min^{-1}; oven temperature program: 1 min isotherm at 50°C followed by a linear temperature increase of 30°C min^{-1} up to 240°C. MS scan conditions: source temperature 230°C, interface temperature 300°C, E energy 70 eV, mass scan range 2–150 amu. The acetone peak was identified by both retention time and mass spectrometry. *E. coli* BL21(DE3)/ pAcetone (acetone biosynthesis) produced acetone while *E. coli* BL21(DE3)/ pET28a did not.

Total ion chromatogram:

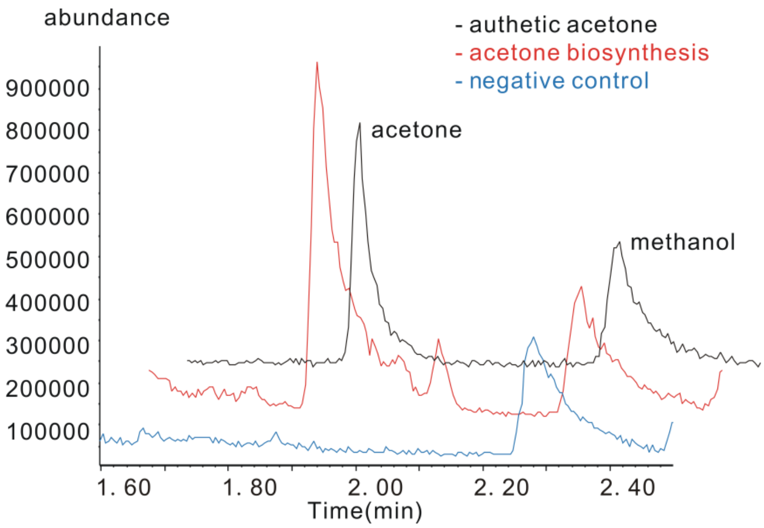

Mass spectra:

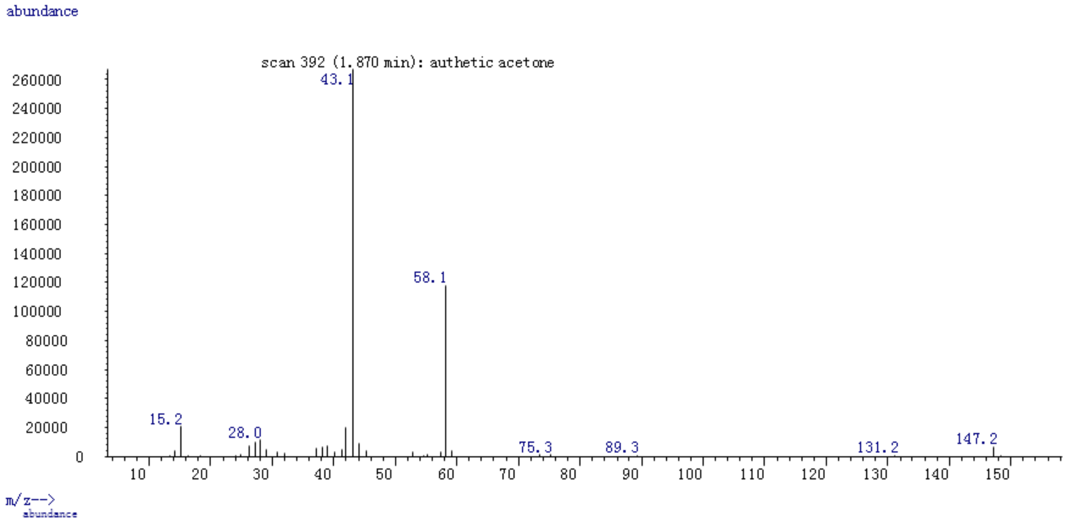

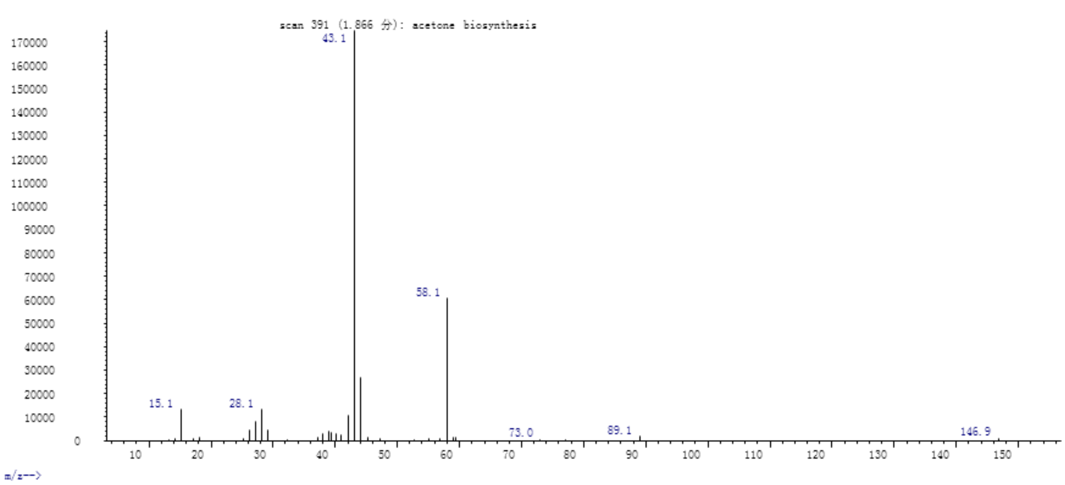

Reference:

1. Bongers RS, Veening J-W, Van Wieringen M, Kuipers OP, Kleerebezem M (2005) Development and Characterization of a Subtilin-Regulated Expression System in Bacillus subtilis: Strict Control of Gene Expression by Addition of Subtilin. Appl Environ Microbiol 71: 8818-8824.
2. Campos N, Rodríguez-Concepción M, Sauret-Güeto S, Gallego F, Lois L, et al. (2001) Escherichia coli engineered to synthesize isopentenyl diphosphate and dimethylallyl diphosphate from mevalonate: a novel system for the genetic analysis of the 2-C-methyl-d-erythritol 4-phosphate pathway for isoprenoid biosynthesis. Biochem J 353: 59.
3. Stratford M, Plumridge A, Archer DB (2007) Decarboxylation of Sorbic Acid by Spoilage Yeasts Is Associated with the PAD1 Gene. Appl Environ Microbiol 73: 6534-6542.
