# Supplementary material for: In Vitro Assembly of Multiple DNA Fragments Using Successive Hybridization
Source: PLoS One. 2012 Jan 26;7(1):e30267. doi: 10.1371/journal.pone.0030267 (PMC3266897; doi:10.1371/journal.pone.0030267)
Supplement: Table S1 — DNA sequencing results. (PDF) [file pone.0030267.s004.pdf]

Table S1 DNA sequencing results

[a]5 clones of pOKCA and 2 clones of pAcetone were sequenced. The other constructs were sequenced for one clone. [b]for pTRICLow and pAcetone only the operon areas were sequenced. The entire plasmids are 9.3 Kb and 6.6 Kb. -, no mutation. Mutations may be introduced from the primers used (primers), or by DNA polymerase during strand extension (PCR). Hybridization itself is not likely to cause any mutation.

Construct (length)	Mutation type	Likely cause	Position and influence
**pOKCA (4.4 Kb ×5[a])**	tttac→ttac (deletion)<br>gttaa→gtaa(deletion)	primers	Not in any genetic element<br>No influence
**pOKC2μUA (7.1 Kb)**	-	-	-
**pOKCA2 (4.4 Kb)**	-	-	-
**pTRICLow (5.1 Kb[b])**	-	-	-
**pJXL (6.3 Kb)**	-	-	-
**pAcetone (3.5Kb[b] ×2[a])**	tgt→tga(transversion)	PCR	In a CDS. Resulted in a premature stop codon
