# Supplementary material for: In Vitro Assembly of Multiple DNA Fragments Using Successive Hybridization
Source: PLoS One. 2012 Jan 26;7(1):e30267. doi: 10.1371/journal.pone.0030267 (PMC3266897; doi:10.1371/journal.pone.0030267)
Supplement: Table S2 — PCR templates and primers for SF preparations. (PDF) [file pone.0030267.s005.pdf]

Table S2

PCR templates and primers for SFs construction.

[a]To reduce transformation backgrounds, plasmids were linearized before using as PCR templates. [b]most SFs were prepared by jointing two DNA fragments together using OE-PCR. In this case there were two templates (the two DNA fragments) in the reaction. In **Supplementary Fig. 1,** the preparation of SF2 is such an example. These intermediate DNA fragments are all highlighted in green. Their preparation (primers and templates used for amplifying them) are also listed in this table. [c]In some cases, a primer has two or more names according to its uses. For example, an anti-sense primer was used to amplify both SF7 and SF3, so it has two names, SF7Wa and SF3Wa. Primer sequences can be found in **Supplementary Table 3**

constructs	PCR products		Templates	Primers	
	SFs	intermediate DNA fragments			
pOKCA (from SF1,2,3,4) and pOKC2μUA(from SF1,2,5,6,7,4)	SF1		pET28a (Novagen) (linearized by HindIII)[a]	SF1Ws	SF1Wa
	SF2		SF2hK+ SF2hC[b]	SF2Ws	SF2Wa
	SF3		SF3hC+ SF3hA[b]	SF3Ws	SF3Wa
	SF4		SF4hA+ SF4hO[b]	SF4Ws	SF4Wa
	SF5		SF5hC +SF5h2μ[b]	SF5Ws	SF5Wa
	SF6		pDR195 (Rentsch et al., 1995)(linearized by HindIII)[a]	SF6Ws	SF6Wa
	SF7		SF7hU +SF7hA[b]	SF7Ws	SF7Wa=SF3Wa[c]
		SF2hK	pET28a (Novagen) (linearized by HindIII)[a]	SF2hKs=SF1Ws[c]	SF2hKa
		SF2hC	pACY duet 1 (Novagen) (linearized by HindIII)[a]	SF2hCs	SF2hCa
		SF3hC	pACY duet 1 (Novagen) (linearized by HindIII)[a]	SF3hCs	SF3hCa
		SF3hA	pKD46 (Datsenko and Wanner, 2000)	SF3hAs	SF3hAa
		SF4hA	pKD46 (Datsenko and Wanner, 2000)	SF4hAs	SF4hAa

		SF4hO	pET28a (Novagen) (linearized by HindIII)[a]	SF4hO	SF4hO=SF1Wa[c]
		SF5hC	pACY duet 1 (Novagen) (linearized by HindIII)[a]	SF5hCs= SF3hCs[c]	SF5hCa
		SF5h2μ	pDR195 (Rentsch et al., 1995)(linearized by HindIII)[a]	SF5h2μs	SF5h2μa=SF6Wa[c]
		SF7hU	pDR195 (Rentsch et al., 1995)(linearized by HindIII)[a]	SF7hUs=SF6Ws[c]	SF7hUa
		SF7hA	pKD46 (Datsenko and Wanner, 2000)	SF7hAs	SF7hAa= SF3hAa[c]
pOKCA2	SFTO		pET28a (Novagen) (linearized by HindIII)[a]	SFTOs=SF4hOs[c]	SFTOa
	SFOT		pET28a (Novagen) (linearized by HindIII)[a]	SFOTs	SFOTa=SF4Wa[c]
	SFTK		pET28a (Novagen) (linearized by HindIII)[a]	SFTKs	SFTKa
	SFKT		pET28a (Novagen) (linearized by HindIII)[a]	SFKTs=SF2Ws[c]	SFKTa=SF2hKa[c]
	SFTC		pACY duet 1 (Novagen) (linearized by HindIII)[a]	SFTCs=SF2hCs[c]	SFTCa
	SFCT		pACY duet 1 (Novagen) (linearized by HindIII)[a]	SFCTs	SFCTa=SF3hCa[c]
	SFTA		pKD46 (Datsenko and Wanner, 2000)	SFTAs=SF3hAs[c]	SFTAa
	SFAT		pKD46 (Datsenko and Wanner, 2000)	SFATs	SFATa=SF4hAa[c]
pTRICLow	SF128		SF128h12+ SF128h8[b]	SF128Ws	SF128Wa
	SF819		SF819h8 +SF819h19[b]	SF819Ws	SF819Wa
	SF19I		SF19I h19+SF19IhI[b]	SF19IWs	SF19IWa
	SFIB		SFIBhI +SFIBhB[b]	SFIBWs	SFIBWa

	SFB		pTrchis2A (Invitrogen)	SFBWs	SFBWa
	SFB12		SFB12hB+ SFB12h12[b]	SFB12Ws	SFB12Wa
		SF128h12	*S. cerevisiae* genomic DNA	SF128h12s	SF128h12a
		SF128h8	*S. cerevisiae* genomic DNA	SF128h8s	SF128h8a
		SF819h8	*S. cerevisiae* genomic DNA	SF819h8s	SF819h8a
		SF819h19	*S. cerevisiae* genomic DNA	SF819h19s	SF819h19a
		SF19I h19	*S. cerevisiae* genomic DNA	SF19I h19s	SF19I h19a
		SF19IhI	*S. cerevisiae* genomic DNA	SF19IhIs	SF19IhIa
		SFIBhI	*S. cerevisiae* genomic DNA	SFIBhIs	SFIBhIa
		SFIBhB	pTrchis2A (Invitrogen)	SFIBhBs	SFIBhBa
		SFB12hB	pTrchis2A (Invitrogen)	SFB12hBs	SFB12hBa
		SFB12h12	*S. cerevisiae* genomic DNA	SFB12h12s	SFB12h12a
pJXL	3SF1		3SF1hD+3SF1hA[b]	3SF1Ws	3SF1Wa
	3SF2		3SF2hA+3SF2hB[b]	3SF2Ws	3SF2Wa
	3SF3		Chemical synthesis	3SF3Ws	3SF3Wa
	3SF4		Chemical synthesis	3SF4Ws	3SF4Wa
		3SF1hD	Chemical synthesis	3SF1hD s=3SF4Ws[c]	3SF1hDa
		3SF1hA	pBR322 (linearized by BamHI)[a]	3SF1hAs	3SF1hAa
		3SF2hA	pBR322(linearized by BamHI)[a]	3SF2hAs	3SF2hAa
		3SF2hB	Chemical synthesis	3SF2hBs	3SF2hBa
pAcetone	SFBT		SFBThB+SFBThT[b]	SFBTWs	SFBTWa
	SFTA		SFTAhT +SFTAhA[b]	SFTAWs	SFTAWa

	SFAH		SFAHhA+SFAHhH[b]	SFAHWS	SFAHWa
	SFHB		SFHBhH+ SFHBhB[b]	SFHBWS	SFHBWa
		SFBThB	pET28aΔ*lacI* (supplementary method2) (linearized by HindIII)[a]	SFBThBs	SFBThBa
		SFBThT	*C. acetobutylicum* ATCC 824 genomic DNA	SFBThTs	SFBThTa
		SFTAhT	*C. acetobutylicum* ATCC 824 genomic DNA	SFTAhTs	SFTAhTa
		SFTAhA	*E. coli* K12 genomic DNA	SFTAhAs	SFTAhAa
		SFAHhA	*E. coli* K12 genomic DNA	SFAHhAs	SFAHhAa
		SFAHhH	*C. acetobutylicum* ATCC 824 genomic DNA	SFAHhHs	SFAHhHa
		SFHBhH	*C. acetobutylicum* ATCC 824 genomic DNA	SFHBhHs	SFHBhHa
		SFHBhB	pET28aΔ*lacI* (supplementary method2) (linearized by HindIII)[a] (supplementary method2)	SFHBhBs	SFHBhBa
Reconstructing pAcetone in a no-gap way	2SFBT		pAcetone (this work)	2SFBTs= SFBTs[c]	2SFBTa
	2SFTA		pAcetone (this work)	2SFTAs	2SFTAa= SFTAa[c]
	2SFAH		pAcetone (this work)	2SFAHs= SFAHs[c]	2SFAHa
	2SFHB		pAcetone (this work)	2SFHBs	2SFHBa= SFHBa[c]
Reconstructing pOKCA with varing	SF4		pOKC2μUA (this work)	SF4Ws	SF4Wa
	SF41		pOKC2μUA (this work)	SF4Ws	SF41Wa=SF3Wa

overlap length	SF42		pOKC2μUA (this work)	SF4Ws	SF42Wa
	SF43		pOKC2μUA (this work)	SF4Ws	SF43Wa
	SF44		pOKC2μUA (this work)	SF4Ws	SF44Wa
	SF2		pOKC2μUA (this work)	SF2Ws	SF2Wa
	SF21		pOKC2μUA (this work)	SF21Ws=SF1 Ws	SF2Wa
	SF22		pOKC2μUA (this work)	SF22Ws	SF2Wa
	SF2		pOKC2μUA (this work)	SF23Ws	SF2Wa
	SF24		pOKC2μUA (this work)	SF24Ws	SF2Wa
