# Supplementary material for: In Vitro Assembly of Multiple DNA Fragments Using Successive Hybridization
Source: PLoS One. 2012 Jan 26;7(1):e30267. doi: 10.1371/journal.pone.0030267 (PMC3266897; doi:10.1371/journal.pone.0030267)
Supplement: Table S3 — Primer sequences. (PDF) [file pone.0030267.s006.pdf]

Table S3

Primer sequences

The sequence of all the primers are provided here.

construct	primer	Primer sequence
pJXL	3SF4Ws	GATCTTTGTCTGCAACTGAAAAGTTT
	3SF4Wa	CTGGAAAAACAAATCGAGTTGTTAA
	3SF3Ws	CAACAAACGAAAATTGGATAAAGTG
	3SF3Wa	ATATTAATTGTTTCTGACTCATCCATCA
	3SF2Ws	GTCAGGTGGCACTTTTCGGG
	3SF2Wa	GTCTCGGACATTCTGCTCCCG
	3SF2hBs	CCGAACGACCGAGCGCAG
	3SF2hBa	ATTTAACTTCTCAGTTTGGCCGCTG
	3SF2hAs	CAAACCGCTAACAATACCTGGG
	3SF2hAa	GGCGCTCTTCCGCTTCC
	3SF1Ws	TCCAGAGGCCTTACAATATGACAC
	3SF1Wa	GGCGCTCTTCCGCTTCC
	3SF1hDa	ATAAACAAATAGGGGTTCCGCG
	3SF1hAs	GTCAGGTGGCACTTTTCGGG
	3SF1hAa	AGCCTGGGGTGCCTAATGAG
pOKCA and pOKC2μUA	SF1Wa	CAAAAATTTAACGCGAAT
	SF1Ws	TTGTTTACCCTCACAACG
	SF2hCa	AAATCTCCGCCCCGTTCGT
	SF2hCs	TGTTAAAATTCGCGTTAAATTTTTGCCTGCCCTGAACCGACGAC

	SF2hKa	GACCCGGTCGTCGGTTCAGGGCAGGCAAAAATTTAACGCGAAT
	SF2Wa	AATGAGACGTTGATCGGCACG
	SF2Ws	GTTCAGCCCGACCGCTGC
	SF3hAa	CGCATGGAAGCGATTAACGAG
	SF3hAs	CTTACGTGCCGATCAACGTCTCATTCGGGGTCTGACGCTCAGTGG
	SF3hCa	TCGTTCCACTGAGCGTCAGACCCCGAATGAGACGTTGATCGGCACG
	SF3hCs	GATCGCTGACGTCGGTACCC
	SF3Wa	AAATGTGCGCGGAACCCC
	SF3Ws	CCTGCCCTGAACCGACGAC
	SF4hAa	ACTGGAACGTTGTGAGGGTAAACAAAAATGTGCGCGGAACCCC
	SF4hAs	ACCCTCGCAAGCTCGGTTG
	SF4hOs	ACAAATAGGGGTTCCGCGCACATTTTTGTTTACCCTCACAACG
	SF4Wa	GGGGGGTTCGTGCACACAG
	SF4Ws	CGGGGTCTGACGCTCAGTGG
	SF6Wa	TCACGAGGCCCTTTCGTCT
	SF6Ws	GAACCTGGGAGTTTTCCCTGA
	SF5hCa	TGTTTCAGGGAAAACTCCCAGGTTCAATGAGACGTTGATCGGCACG
	SF5h2μs	CTTACGTGCCGATCAACGTCTCATTGAACCTGGGAGTTTTCCCTGA
	SF5Wa	ATACGATACCCCGCATGGAATG
	SF7hUa	TCGTTCCACTGAGCGTCAGACCCCGTCACGAGGCCCTTTCGTCT
	SF7hAs	TCTTGAAGACGAAAGGGCCTCGTGACGGGGTCTGACGCTCAGTGG
	SF7Ws	GCTTCCTTCAGCACTACCCTTTAG
pOKCA from 8 SFs	SFTOa	TGAGATACCTACAGCGTGAG
	SFTKs	GTTCGGTGTAGGTCGTTCG
	SFTKa	AAATATTAACGTTTACAATTTCAGG
	SFTCa	AGGTTCCAACTTTCACCATAATGAA

	SFTAa	TTATTTTTCTAAATACATTCAAATATGTATCCGC
	SFOTs	AACCGGGCATGTTCATCATCA
	SFCTs	GAATTTGCTTTCGAATTTCTGCC
	SFATs	AAACTCACGTTAAGGGATTTTGGTC
pTRICLow	SF128Ws	TCATTACCGTTCTTAACTTCTGCAC
	SF128Wa	ATTCTGCATGCAGCTACCTTAAGTTATTTATCAAGATAAGTTTCCGGATC
	SF819Ws	ACCCTTAAGGAGGAAAAAAACATGTCAGAGTTGAGAGCCTTCA
	SF819Wa	GCGATGCAGCGAATTGATCTTATTCCTTTGGTAGACCAGTCTT
	SF19IWs	TCGCCCTTAAGGAGGAAAAAAAAATGACCGTTTACACAGCATCCG
	SF19IWa	TTATAGCATTCTATGAATTTGCCTGTC
	SFIBWs	CCTTAGGAGGTAAAAAAAAATGACTGCCGACAACAATAGTATGCC
	SFIBWa	CACGAACCCCCCGTTCAGC
	SFBWs	CTGCAGCTGGTACCATATGG
	SFBWa	CATGGTTTATTCCTCCTTATTTAAT
	SFB12Ws	CACACAGCCCAGCTTGG
	SFB12Wa	GCAGGCCTATCGCAAATTAGCTTATGAAGTCCATGGTAAATTCG
	SF128h12s	CTCCCGTGGAAAGCTAGCG
	SF128h12a	CATGTTTTTTTCCTCCTTAAGGGTGCAGGCCTATCGCAAATTAGCTTATGAAGTCCATGGTAAATTCGTG
	SF128h8s	TAAGCTAATTTGCGATAGGCCTGCACCCTTAAGGAGGAAAAAAACATGTCAGAGTTGAGAGCCTTCAGT
	SF128h8a	AGATTTTGCCCAAGCCCG
	SF819h8s	AGAAGGCAGTTCTGCCGCA
	SF819h8a	TTTTTTTTCCTCCTTAAGGGCGAATTCTGCATGCAGCTACCTTAAGTTATTTATCAAGATAAGTTTCCGGATC
	SF819h19s	CTTAAGGTAGCTGCATGCAGAATTCGCCCTTAAGGAGGAAAAAAAAATGACCGTTTACACAGCATCCG
	SF819	TGTTACAGCCGCGTTTTGAC

	h19a	
	SF19IhIs	AATAAGATCAATTCGCTGCATCGCCCTTAGGAGGTAAAAAAAAATGACTGCCGACAACAATAGTATG
	SF19IhIa	TGGTGGGTTTGTGGCCG
	SF19Ih19s	GCCTCATCAGCTCCGCATA
	SF19Ih19a	GTCATTTTTTTTACCTCCTAAGGGCGATGCAGCGAATTGATCTTATTCCTTTGGTAGACCAGTCTTTG
	SFIBhIs	CATGCGGCTATTTTGCCTAT
	SFIBhIa	AATTCCCATATGGTACCAGCTGCAGTTATAGCATTCTATGAATTTGCCTG
	SFIBhBs	CAGGCAAATTCATAGAATGCTATAACTGCAGCTGGTACCATATGGG
	SFIBhBa	CACGTAGCGATAGCGGAGTG
	SFB12hBs	TTCAAGAACTCTGTAGCACC
	SFB12hBa	GCAGAAGTTAAGAACGGTAATGACATGGTTTATTCCTCCTTATT
	SFB12h12s	ATTAAATAAGGAGGAATAAACCATGTCATTACCGTTCTTAAC
	SFB12h12a	GGTCTGCTTAAATTTCATTC
pAcetone	SFAHa	ATCATATATAACTTCAGCTCTAGGC
	SFAHhAa	TTCATCCTTTAACATATGTATATCTCCTTCTCATAAATCACCCCGTTGC
	SFAHhAs	AACTCCAGGAATACGGTATCG
	SFAHhHa	ACAGAACTTGGAGTAATTGAGG
	SFAHhHs	CGGGGTGATTTATGAGAAGGAGATATACATATGTTAAAGGATGAAGTAATTAAAC
	SFAHs	TGATGACATTACAAGACGCC
	SFBTa	TTGTCCGCCACCTATACATAAAGTTG
	SFBThBa	CTGCACTAGCTATTACAACTTCTTTCATGGTATATCTCCTTCTTAAAGTT
	SFBThBs	GTGATGCACCTGGACCTG
	SFBThTa	TTTTAGGCCATTTCTTATCGC

	SFBThTs	TAACTTTAAGAAGGAGATATACCATGAAAGAAGTTGTAATAGCTAGTGC
	SFBTs	TCACCGCGGAAATGGCG
	SFHBa	CACAATTCCCCTATAGTGAGTCGTA
	SFHBhBa	GGCAACTGCACGGTGGAGG
	SFHBhBs	AGTTATATATGATTATCTTAAGTAATCACCGCGGAAATGGCG
	SFHBhHa	TCGCCCACCGCCATTTCCGCGGTGATTACTTAAGATAATCATATATAACTTCAGC
	SFHBhHs	TGAATTCATAAAAACACCTCCAC
	SFHBs	TAGCACGCCATTAACTTCGC
	SFTAa	CCCCGTTGCGTATTCAG
	SFTAhAa	GCAGTACGCAGCAAACTTTTC
	SFTAhAs	CTAGAAAAGTGCTAGGAAGGAGATATACATATGAAAACAAAATTGATGACATTAC
	SFTAhTa	CAATTTTGTTTTCATATGTATATCTCCTTCCTAGCACTTTTCTAGCAATATTGC
	SFTAhTs	GGGGGTTAGCATATGCATAAG
	SFTAs	GGAAAGTCTCTTAAGGATGTACC
pAcetone no-gap reconstruction	2SFAHa	TTACTTAAGATAATCATATATAACTTCAGC
	2SFBTa	ATTTTGTTTTCATATGTATATCTCCTTCCTAGC
	2SFHBs	TGATTTATGAGAAGGAGATATACATATGTTA
	2SFTAs	AGCGGATAACAATTCCCC
Reconstructing pOKCA with varing overlap length	SF4Ws	CGGGGTCTGACGCTCAGTGG
	SF4Wa	GGGGGGTTCGTGCACACAG
	SF41Wa	AAATGTGCGCGGAACCCC
	SF42Wa	AACGTTGTGAGGGTAAACAAAAATGTG

	SF43Wa	TGAACATGCCCGGTTACTGGAA
	SF44Wa	GGTGCACTCTCAGTACAATCTGCTCTG
	SF2Ws	GTTCAGCCCGACCGCTGC
	SF21Ws	TTGTTTACCCTCACAACG
	SF22Ws	CCAGTAACCGGGCATGTTCAT
	SF23Ws	TCATCAGTAACCCGTATCGTGAGC
	SF24Ws	ATATATGCGGTGTGAAATACCGCAC
	SF2Wa	AATGAGACGTTGATCGGCACG
