# Supplementary material for: In Vitro Assembly of Multiple DNA Fragments Using Successive Hybridization
Source: PLoS One. 2012 Jan 26;7(1):e30267. doi: 10.1371/journal.pone.0030267 (PMC3266897; doi:10.1371/journal.pone.0030267)
Supplement: Table S4 — Construction of pAcetone by SLIC. (PDF) [file pone.0030267.s007.pdf]

# Table S4

Construction of pAcetone by SLIC.

4 fragments were designed to have 50 bp homologous ends. Repeated sequences (15 bp RBS+3 bp ATG) is highlighted in red. Vector backbone was amplified from pET28aΔlacI. Insert fragments *thl atoAD* and *adc* were generated by PCR. Then they were purified, treated with T4 DNA polymerase and assembled by SLIC with the help of recA.

| fragments | templates | primers | |
|---|---|---|---|
| *thl* | *Clostridium acetobutylicum* genomic DNA | thl s | thl a |
| *atoAD* | *E. coli* K12 genomic DNA | atoAD s | atoAD a |
| *adc* | *Clostridium acetobutylicum* genomic DNA | adc s | adc a |
| vector | pET28aΔ*lacI* | B s | B a |
| Primer sequences | | | |
| thl s | TAACTTTAAGAAGGAGATATACCATGAAAGAAGTTGTAATAGCTAGTGC | | |
| thl a | CAATTTTGTTTTCATATGTATATCTCCTTCCTAGCACTTTTCTAGCAATATTGC | | |
| atoAD s | CTAGAAAAGTGCTAGGAAGGAGATATACATATGAAAACAAAATTGATGACATTAC | | |
| atoAD a | TTCATCCTTTAACATATGTATATCTCCTTCTCATAAATCACCCCGTTGC | | |
| adc s | CGGGGTGATTTATGAGAAGGAGATATACATATGTTAAAGGATGAAGTAATTAAAC | | |
| adc a | TCGCCCACCGCCATTTCCGCGGTGATTACTTAAGATAATCATATATAACTTCAGC | | |
| B s | AGTTATATATGATTATCTTAAGTAATCACCGCGGAAATGGCG | | |
| B a | CTGCACTAGCTATTACAACTTCTTTCATGGTATATCTCCTTCTTAAAGTT | | |
