# Supplementary material for: In Vitro Assembly of Multiple DNA Fragments Using Successive Hybridization
Source: PLoS One. 2012 Jan 26;7(1):e30267. doi: 10.1371/journal.pone.0030267 (PMC3266897; doi:10.1371/journal.pone.0030267)
Supplement: Method S1 — Cloning Populus alba isoprene synthase. (PDF) [file pone.0030267.s008.pdf]

Method S1

Cloning *Populus alba* isoprene synthase

The 105bp N' end of the original mRNA (GenBank: AB198180.1) coding a transit peptide was omitted. The remaining sequence was codon-optimized for better expression in *E. coli* using an on-line tool, OPTIMIZER (http://genomes.urv.es/OPTIMIZER/), and synthesized chemically. Then it was amplified using primers ispSAs (5'-GGAATTCCATATG[NdeI]AGATGTAGCGTGTCCACCGAA-3') and ispSAa (5'-GGAAGATCT [BglII] TTAGCGTTCAAACGGCAGAATC-3'). The PCR product and pACYduet1 were digested with NheI and BglII and subsequently ligated together. Ligation mixtures were transformed into *E. coli* DH5a chemical component cells, and the resulting transformants were plated on LB agar with chloramphenicol.
