# Supplementary material for: In Vitro Assembly of Multiple DNA Fragments Using Successive Hybridization
Source: PLoS One. 2012 Jan 26;7(1):e30267. doi: 10.1371/journal.pone.0030267 (PMC3266897; doi:10.1371/journal.pone.0030267)
Supplement: Method S2 — Construction of pET28aΔ lacI . (PDF) [file pone.0030267.s009.pdf]

method S2

Construction of pET28aΔ*lacI*

pET28a was amplified with primers pET28aΔ*lacI*s (5'-GCCCAGTAGTAGGTTGAGGC-3') and pET28aΔ*lacI*a (GAAGATCT BglII TCGGTTTCCGTGTTTCGTA). The PCR product was gel-purified and digested with BglII, and self-ligated (one BglII site existed on pET28a, the other was introduced by primer pET28aΔ*lacI*a). In this way we deleted the *lacI* from pET28a and made pET28aΔ*lacI*. Ligation mixtures were transformed into *E. coli* DH5a chemical component cells, and the resulting transformants were plated on LB agar with kanamycin.
