# Supplementary material for: In Vitro Assembly of Multiple DNA Fragments Using Successive Hybridization
Source: PLoS One. 2012 Jan 26;7(1):e30267. doi: 10.1371/journal.pone.0030267 (PMC3266897; doi:10.1371/journal.pone.0030267)
Supplement: Method S3 — Reconstructing pAcetone in a no-gap way. (PDF) [file pone.0030267.s010.pdf]

method S3

Reconstructing pAcetone in a no-gap way

In order to evaluate the impact of long gaps on the assembly efficiency, we parallelly carried out two constructions. One was repeating the construction described in the text (section Long gaps tolerated by SHA) which would generate gaps of 110, 54, 58 and 12 nt. The other was basically the same with the first one, except that primers SFTAs, SFHBs, SFBTa, SFAHa were replaced by 2SFTAs, 2SFHBs, 2SFBTa, 2SFAHa, such that nicks instead of gaps would form. Transformations were plated on plates containing kanamycin. The difference between them is schematically illustrated:

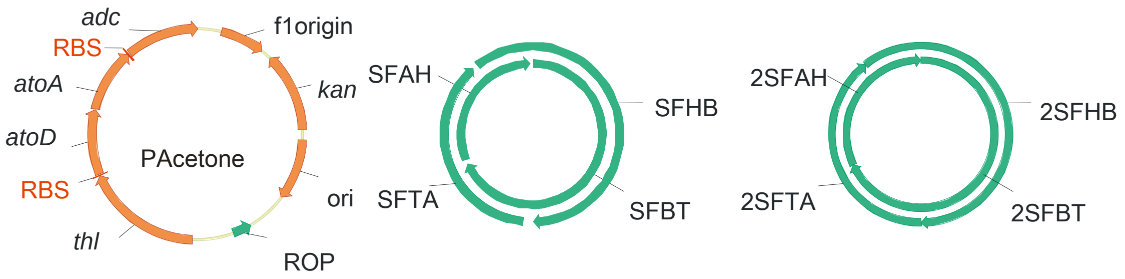

Plasmid map Gap way No-gap way
